# Markov Networks of Collateral Resistance: National Antimicrobial Resistance Monitoring System Surveillance Results from *Escherichia coli* Isolates, 2004-2012

**DOI:** 10.1371/journal.pcbi.1005160

**Published:** 2016-11-16

**Authors:** William J. Love, Kelson A. Zawack, James G. Booth, Yrjo T. Grӧhn, Cristina Lanzas

**Affiliations:** 1 Department of Population Health and Pathobiology, College of Veterinary Medicine, North Carolina State University, Raleigh, North Carolina, United States of America; 2 Department of Biological Statistics & Computational Biology, College of Agriculture and Life Sciences, Cornell University, Ithaca, New York, United States of America; 3 Tri-Institutional Program in Computational Biology & Medicine, New York City, New York, United States of America; 4 Department of Population Medicine and Diagnostic Sciences, College of Veterinary Medicine, Cornell University, Ithaca, New York, United States of America; University of New South Wales, AUSTRALIA

## Abstract

Surveillance of antimicrobial resistance (AMR) is an important component of public health. Antimicrobial drug use generates selective pressure that may lead to resistance against to the administered drug, and may also select for collateral resistances to other drugs. Analysis of AMR surveillance data has focused on resistance to individual drugs but joint distributions of resistance in bacterial populations are infrequently analyzed and reported. New methods are needed to characterize and communicate joint resistance distributions. Markov networks are a class of graphical models that define connections, or edges, between pairs of variables with non-zero partial correlations and are used here to describe AMR resistance relationships. The graphical least absolute shrinkage and selection operator is used to estimate sparse Markov networks from AMR surveillance data. The method is demonstrated using a subset of *Escherichia coli* isolates collected by the National Antimicrobial Resistance Monitoring System between 2004 and 2012 which included AMR results for 16 drugs from 14418 isolates. Of the 119 possible unique edges, 33 unique edges were identified at least once during the study period and graphical density ranged from 16.2% to 24.8%. Two frequent dense subgraphs were noted, one containing the five β-lactam drugs and the other containing both sulfonamides, three aminoglycosides, and tetracycline. Density did not appear to change over time (p = 0.71). Unweighted modularity did not appear to change over time (p = 0.18), but a significant decreasing trend was noted in the modularity of the weighted networks (p < 0.005) indicating relationships between drugs of different classes tended to increase in strength and frequency over time compared to relationships between drugs of the same class. The current method provides a novel method to study the joint resistance distribution, but additional work is required to unite the underlying biological and genetic characteristics of the isolates with the current results derived from phenotypic data.

## Author Summary

Surveillance of antimicrobial resistance patterns is an important responsibility in modern public health. Due to the genetic configuration of bacterial pathogens, the use of a single antimicrobial drug may select for bacteria that are resistant to multiple other antimicrobial drugs via set of mechanisms collectively known as “collateral resistance”. We have developed a new analytic method to study and describe possible collateral resistance pathways using existing antimicrobial resistance surveillance data. The method, named “R-nets”, use network models to visualize the patterns of collateral resistance which allows large amounts of information to be clearly communicated to public health officials and researchers. Applying the R-nets to publicly available data from *E*. *coli* collected by the FDA and USDA between 2004 and 20012 we found that the number of collateral resistance links was relatively constant, but there may be an increase in collateral resistances between drugs of different structures. The method described will hopefully be useful for predicting and managing the proliferation of highly resistant bacterial pathogens

## Introduction

The evolution of acquired antimicrobial resistance (AMR) in pathogenic microorganisms is one of the foremost challenges in public health today. Antimicrobial drug use in medicine and agriculture generates selective pressure that selects for AMR in bacterial populations and facilitates emergence of multiple drug resistant (MDR) phenotypes [1]. Bacterial pathogens with an MDR phenotype pose a substantial clinical challenge since the antimicrobial drugs typically prescribed may not effectively clear a patient’s infection and delay the patient’s recovery. The most recent and dramatic example is the emergence of plasmid-mediated resistance to colistin in *Escherichia coli* isolated from animals and humans [2]. Colistin is the last resort to treat some infections caused by Gram-negative bacteria such as carbapenem resistant *Actinobacter baumannii* and at least one pan-resistant strain of *E*. *coli* has been isolated from a clinically ill human patient [3, 4].

Multiple mechanisms of bacterial evolution, including mutation, recombination, and clonal expansion, give rise to highly resistant strains of bacteria and encourage persistence of these strains following their emergence [5]. Genetic capitalism describes the phenomenon by which the progeny of a microbe with one fitness trait, a drug-resistant phenotype for example, tend to survive serial selection events, in turn increasing the likelihood that the progeny will acquire additional fitness traits via recombination [6]. The initial fitness trait may be acquired by mutation, horizontal gene transfer (HGT), or novel recombination event with other bacteria in the environment. An example of genetic capitalism was the rapid emergence and expansion of a fluoroquinolone-resistant strain of methicillin-resistant *Staphylococcus aureus* (MRSA) at the Atlanta Veterans Affairs Medical Center, where the proportion of fluoroquinolone-resistant MRSA isolates increased from 0 to nearly 80% within 1 year of the introduction of ciprofloxacin to the hospital's formulary [7]. Collateral selection, another mechanism capable of generating MDR strains, describes the phenomenon where selection pressure from one antimicrobial drug may additionally select for or against phenotypic resistances to other drugs via several mechanisms [8]. Cross-resistance describes resistance to several related drugs by a single mechanism, e.g., point mutations to the DNA gyrase subunit A gene (*gyrA*) that increase resistance to quinolone antibiotics [9], co-resistance describes a set of resistances conferred by a set of frequently concurrent genes, e.g., polymixin and colistin resistance genes carried on the recently-described MCR-1 plasmid in *Escherichia coli* [2], and pleiotropic resistance describes individual mutations that simultaneously affect resistances for multiple unrelated drugs such as *marR* gene alterations in *E*. *coli* that increase resistance to tetracycline, chloramphenicol, and fluroquinolones drugs [10, 11].

Monitoring the proliferation of existing MDR strains and emergence of novel MDR strains are primary goals of AMR surveillance programs carried out by governmental agencies across the world including, but not limited, to the Department of Agriculture (USDA), Food and Drug Administration (FDA) and Centers for Disease Control and Prevention (CDC) in the United States [12–14], the Department of Health in the United Kingdom [15], the European Centers for Disease Control and Prevention (ECDC) in the European Union [16, 17], and the collaborative Transatlantic Task Force on Antimicrobial Resistance created in 2009 [18, 19]. These agencies' survey methodologies and their published reports have been largely focused on univariate phenotypic resistances and the prevalence of MDR strains [20]. These surveillance reports however provide little information about the joint distributions of drug resistances in the overall population that may contribute to MDR strain development and emergence via collateral resistance. Outside of AMR surveillance, several studies of *in vitro* and *in situ* bacterial populations have begun to explore joint resistance distributions in an effort to identify strategies to mitigate MDR development [21–23].

Studying joint distributions of resistances in AMR surveillance data is necessary to understand *in situ* MDR strain evolution, but poses a number of challenges. The number of estimated correlation coefficients required to fully describe the pair-wise resistance distributions grows quadratically with the number of drugs in a panel, specifically _*k*_*C*_2_ = *k*^2^/2 –*k*/2 for a panel of *k* drugs; Most AMR panels contain a dozen drugs or more, requiring at least _*12*_*C*_2_ = 66 correlations to be estimated. Hypothesis testing may be used to determine which drug pairs are not correlated, but the large number of estimated correlations results substantial type I error rate inflation. Pair-wise correlations also do not control for confounding by other variables in the dataset.

Better methods are needed to characterize and quantify the joint resistance distributions in bacteria. We propose graphical models, specifically Markov networks, to study joint distributions in AMR surveillance. Graphical models are mathematical constructs which represent the interactions between elements in complex systems. A variety of well-studied parameters, e.g., density and modularity, have been described to summarize these models at multiple levels of complexity. This study’s objective is to estimate the Markov networks' structures that represent existing AMR surveillance data to effectively describe and visualize AMR relationships among a population of bacteria. This technique is intended to supplement the current methods used to analyze and report AMR surveillance results. The method is demonstrated here using data collected by the National Antimicrobial Resistance Monitoring System for Enteric Bacteria (NARMS).

## Materials and Methods

### Surveillance data

The NARMS study tracks AMR in *E*. *coli*, *Salmonella* spp., *Enterococcus* spp., and *Campylobacter* spp. isolated from slaughter houses, retail meat, and cases of food borne illness by the USDA, FDA and CDC respectively. Surveillance results from NARMS between 1998 and 2013 were recently made publically available [24]. Isolates were tested for AMR with genus-specific drug panels, typically containing 13 to 15 drugs with resistance results reported as minimum inhibitory concentrations (MIC).

To demonstrate our approach, a subset of the NARMS data was selected from the AMR results from *E*. *coli* isolated between 2004 and 2012 (*n* = 14,418). The MIC results for the following 16 of the available 23 drugs were used to demonstrate the networks: ampicillin (AMP), amoxicillin and clavulanic acid (AMC), ceftriaxone (AXO), cefoxitime (FOX), ceftiofur (TIO), amikacin (AMI), gentamicin (GEN), kanamycin (KAN), streptomycin (STR), nalidixic acid (NAL), ciprofloxacin (CIP), sulfisoxazole (FIS), trimethoprim and sulfamethoxazole (COT), chloramphenicol (CHL), tetracycline (TET), and azithromycin (AZI). The drug resistances were grouped into classes based on the structure of the drug tested (Table 1). The breakpoints for these antimicrobials and summary of observed resistances in the NARMS data are provided in Table 2.

**Table 1:** Descriptions of 16 antimicrobial drugs included in the AMR panel for *E*. *coli* isolates from chicken carcass rinsates and commercial chicken breasts between 2004 and 2012 collected by the NARMS study.

Drug	Abbrev.	Class	Target Mechanism (Targets)
Ampicillin	AMP	β-lactams	Cell Wall Synthesis (Penicillin Binding Protein)
Amoxicillin + Clavulanic Acid	AMC		
Ceftriaxone	AXO		
Cefoxitime	FOX		
Ceftiofur	TIO		
Amikacin*	AMI	Aminoglycosides	Protein Synthesis (30S Ribosomal Subunit)
Gentamicin	GEN		
Kanamycin	KAN		
Streptomycin	STR		
Ciprofloxacin	CIP	Quinolones	DNA Synthesis Inhibition
Nalidixic Acid	NAL		(Topoisomerase IV & Gyrase)
Trimethoprim + Sulfamethoxazole	COT	Sulfonamides	Folic Acid Synthesis (Dihydropteroate Synthase)
Sulfisoxazole	FIS		
Tetracycline	TET	Tetracyclines	Protein Synthesis
			(30S Ribosomal Subunit)
Chloramphenicol	CHL	Phenicols	Protein Synthesis
			(50S Ribosomal Subunit)
Azithromycin†	AZI	Macrolides	Protein Synthesis
			(50S Ribosomal Subunit)

*Amikicin was only included in the drug panels during 2004–10, and removed afterward.

†Azithromycin was included in panels during 2011 & 2012

**Table 2:** Summary of AMR testing ranges, resistance breakpoints, and results from 14,418 *E*. *coli* isolates collected from chicken carcass rinsates and commercial chicken breasts between 2004 and 2012 to an AMR panel of 16 drugs by the NARMS study. The breakpoints were collected from CLSI and USDA-ARS documents [57, 63].

Drug	Min. Obs MIC*	Max. Obs MIC	Dil'ns Obs	Range Tested(# Dil'ns)		MIC Breakpt.	Min Res. (%)	Max Res. (%)	Mean % Res.
AMP	≤ 1	> 32	7	1	- 32 (6)	≥ 32	17.2	24.3	20.5
AMC	≤ 1	> 32	7	1	- 32 (6)	≥ 32*	5.6	14.9	10.7
AXO	≤ 1/4	> 64	10	1/4	- 64 (9)	≥ 4	4.4	13.4	9.8
FOX	≤ 1/2	> 32	8	1/2	- 32 (7)	≥ 32	5.0	14.1	10.3
TIO	≤ 1/8	> 8	8	1/8	- 8 (7)	≥ 8	4.4	10.5	7.9
AMI	≤ 1/2	≤ 16	6	1/2	- 64 (8)	≥ 64	0.0	0.0	0.0
GEN	≤ 1/4	> 16	8	1/4	- 16 (7)	≥ 16	30.8	42.0	38.0
KAN	≤ 8	> 64	5	8	- 64 (4)	≥ 64	5.7	10.6	8.4
STR	≤ 32	> 64	3	32	- 64 (2)	≥ 64	38.9	62.7	49.0
CIP	≤ 1/64	> 4	10	1/64	- 4 (9)	≥ 1†	0.1	0.6	0.3
NAL	≤ 1/2	> 32	8	1/2	- 32 (7)	≥ 32	2.3	7.4	4.2
COT	≤ 1/8	> 4	6	1/8	- 4 (6)	≥ 4*	3.1	9.9	6.7
FIS	≤ 16	> 256	6	16	- 256 (5)	≥ 512	39.2	51.4	47.5
TET	≤ 4	> 32	5	4	- 32 (4)	≥ 16	40.2	49.8	45.5
CHL	≤ 2	> 32	6	2	- 32 (5)	≥ 32	0.9	2.2	1.4
AZI	≤ 1/2	> 16	7	1/8	- 16 (8)	≥ 32	0.0	0.4	0.2

*Reported breakpoint for primary drug in combination: Amoxicillin in AMC and Sulfamethoxazole in COT

†Reported breakpoints conflict between those reported by CLSI (≥ 4) and the USDA-ARS website (≥ 1). The USDA-ARS breakpoint was used to define in the current study.

Additional information regarding the data and sample sizes is available in supplemental material (S1 Text).

### Statistical analysis

All statistical analyses were performed using R version 3.2.3 [25]. Spearman's rank correlations were used to estimate MIC relationships and trends in graphical parameters over time [26]. Sparse graphical model structures were constructed using the glasso package version 1.8 [27] and graphical parameters were estimated using the igraph package version 1.0.1 [28].

### Overview of network inference

A graphical model *G* of a system is comprised two sets: the vertex set *G(V)* which defines the system’s *k* discrete elements and the edge set *G(E)* which defines the *m* pair-wise interactions between the system’s elements. For simple graphical models, *G(E)* may be expressed as the set of unique, unordered pairs of adjacent vertices (*G(E)* = {(*v*_*i*_, *v*_*j*_)| *v*_*i*_ adjacent to *v*_*j*_, *i* ≠ *j*} or as an adjacency matrix [29].

A Markov network is a specific type of undirected graphical model used to represent relationships between variables in a data set [30, 31]. Each of the dataset’s *k* constituent random variables are represented by a vertex in the Markov network (*G(V)* = {*v*_*i*_, …, *v*_*k*_}). Edges in a Markov network are defined by the variables' partial correlations (*ω*_*ij*_), which represent the standardized covariance of a pair of variables *v*_*i*_ and *v*_*j*_ when conditioned on all other variables in the dataset. When *ω*_*ij*_ = 0, *v*_*i*_ and *v*_*j*_ are conditionally independent and are not adjacent in a Markov network. Therefore, a Markov network's edge list consists of the *m* unique, unordered pairs of variables which are not conditionally independent (*G(E)* = {(*v*_*i*_, *v*_*j*_)| *ω*_*ij*_ ≠ 0}) and the network’s adjacency matrix **A** may be defined from **Ω** using the indicator function 𝟙_M_ (Eq 1).

$$A_{ij}={𝟙}_{M}\left(\omega_{ij}\right)=\left[\omega_{ij}\neq 0\right]$$ Eq 1

When the structure of a system's Markov network is unknown, it is possible to estimate the partial correlations and network structure from observed data. An empirical correlation matrix **Σ** can be inverted to produce an precision matrix **Θ** (**Θ** = **Σ**^-1^), which in turn can be used to estimate **Ω** (Eq 2) [32].

$$\omega_{ij}=\frac{-\vartheta_{ij}}{\sqrt{\vartheta_{ii}\vartheta_{jj}}}$$ Eq 2

Graphical models estimated in this way are typically complete (*m =*_*k*_*C*_*2*_) since trivial correlations will persist, even after conditioning on the other variables in the set. However, graphical models, including Markov networks, are easier to interpret and are more useful when they are sparse (*m* << _*k*_*C*_*2*_) [31].

The least absolute shrinkage and selection operator (LASSO) is a method used to generate more parsimonious models of joint distributions. The graphical LASSO applies an L_1_-regularization penalty *ρ* to estimate a penalized precision matrix (Θ*) [27]. The penalization reduces the absolute value of all elements of **Θ** (|*ϑ*_*ij*_| > |ϑ^*^_ij_|) and if the penalty is large enough, some *ϑ*_ij_^*^ are reduced to zero. When estimated from Θ* instead of **Θ**, the trivially small partial correlations are reduced to zero and the Markov network will be sparse. While any non-negative value may be assigned to *ρ*, its useful range is limited to *min*|*ϑ*_*ij*_|< *ρ* < *max*(|*ϑ*_*ij*_|). When *ρ* > *max*(|*ϑ*_*ij*_|), Θ* and **Ω** are diagonal, all *ω*_*ij*_ are trivial, and *G(E)* = ∅; conversely, if *ρ* < *min*(|*ϑ*_*ij*_|), then no *ω*_*ij*_ are trivial, and the resultant graphical model will typically be complete, similar to using the unpenalized precision matrix.

### Graphical models of collateral resistance

The Markov networks of collateral resistance developed here are subsequently referred to as "R-nets" and two versions are presented: simple R-nets (*R*) and weighted R-nets (*R’*). For these networks, *R(V)* = *R’(V)* and *R(E)* = *R’(E)*, but their adjacency matrices are different. For *R*, the simple adjacency matrix **A** is defined by Eq 1. For *R’*, **Ω** is used as the weighted adjacency matrix where the elements *ω*_*ij*_ represent the strength and direction of the relationship. The vertices of an R-net represent the observed distribution antimicrobial resistances, e.g., TIO represents the observed set of MIC_TIO_. The edges of an R-net represent correlated resistances, which in turn identify potential collateral resistances in the population. A Spearman's rank correlation matrix **Σ**_*y*_ was estimated for the adjusted log_2_MIC values within each year *y*. A non-parametric estimator was used because the distribution of the MICs, even when transformed, frequently did not conform to a normal distribution, and Pearson’s correlation estimates may yield biased results when applied to non-normal data [26]. The graphical LASSO and Eq 2 were used to estimate the structures of *R*_*y*_ and *R’*_*y*_. The graphical LASSO method assumes multivariate normal data, but it has been demonstrated that an untransformed Spearman’s correlation matrix may be substituted for the more traditional parametric correlation matrix, provided the former is positive semidefinite [33].

### Regularization penalty selection

The structures of sparse Markov networks generated via the graphical LASSO are conditional on the L_1_-penalty used to estimate Θ^*^, thus the selection of the penalty is an important step in the inference process. In general, the value of ρ should be low enough to assure no important edges are lost, and high enough to effectively reduce the density of the graph. Comparisons of *R*_*y*_ and *R’*_*y*_ over time were restricted to sets of R-nets generated from a common penalty to improve comparability of the networks. To select a common penalty value, 12 sets of *R* and *R’* were produced and evaluated with each set of networks generated using a separate value of *ρ* between 0.05 and 0.60. The structure and distribution of density (${\bar{m}}_{y}$; Eq 3) within each set of R-nets were subjectively evaluated to select a single penalty value to apply across all years. The common penalty was selected via a supervised review of the R-nets based on network interpretability and trends in ${\bar{m}}_{y}$ over *ρ*. A network should be sparse enough to be understood when visualized, meaning the graph should not be too dense for a viewer to understand the set of edges present. Edges representing expected cross-resistances, e.g., NAL-CIP, AMP-AMC, and FIS-COT, should be strong and frequently present. Additionally, the number of unstable cycles (cycles with an odd number of negative edges) should be minimized [34]. The median density (${\bar{m}}_{50}$) and range of ${\bar{m}}_{y}$ was plotted against the evaluated values of *ρ* and a point was sought where the slope of the line broke from steeply decreasing to level off; this method is similar to the Scree test for factor retention in FA and PCA [35, 36]. The lowest penalty that generated *R* and *R’* that met these criteria were selected for further evaluation.

$${\bar{m}}_{y}=\frac{m_{y}}{{}_{k}C{}_{2}}$$ Eq 3

### Longitudinal comparison of graphical parameters

Three graphical parameters were used to evaluate changes in AMR resistance over time described by *R* and *R'*: density, vertex degree, and modularity. Density quantifies a graph's overall interconnectivity and trends in $\bar{m}$ could indicate overall trends in AMR relationships and risk of collateral resistance in the population over time [37]. Increasing density over time represents more interconnectivity of drug resistances.

Vertex degree *d*_*i*_ is equal to the number of other vertices adjacent to *v*_*i*_. In an R-net, *d* describes the number of other resistances one MIC is related to in the population. In the R-nets vertices with high *d*_*i*_ may represent resistances which are influential to the development of MDR strains. For example, if resistance to drug A had a high degree in a bacterial population, e.g., d_A_ = 5, the use of drug A could select for increased resistance to A and potentially affect resistance to five other drugs, assuming that the covariance structure of phenotypic drug resistance identified by the Markov networks reflects genetic mechanisms allowing for collateral resistance. The high vertex degree of A does not indicate that the drug resistance is responsible for resistance noted in five adjacent vertices, only that selection for A could possibly influence the other resistances.

Modularity (*Q*) measures how frequently adjacent vertices are similar or dissimilar as defined by vertex attributes [38]. The class of drug associated with the resistance of each vertex was used to assign group membership (Table 1). Modularity is positive when edges join similar vertices more frequently than would be expected by chance and negative when dissimilar vertices are more frequently than would be expected by chance. Trends in modularity may represent shifting tendencies in resistance relationships to exist between more similar or less similar drugs. Similar estimates of modularity for signed and weighted networks (*Q'*) can be estimated [39]. The value of *Q'* describes the relative strength of edges between similar vertices compared to dissimilar vertices. Spearman's rank correlation was used to test for a trends in $\bar{m}$, *Q*, and *Q'* over time due to the small number of values being compared [26].

### Principal component analysis

Principal component analysis has been used to study resistance relationships and was performed here to evaluate how the current method compares to a previously employed method [23]. Three years of the study were selected to represent data from the beginning, middle, and end of the study, respectively, and PCA was performed separately on the data from each year. The eigenvalues of **Σ**_*y*_ (*λ*) were computed and the components for which *λ* > 1 were extracted and subsequently oblimin rotated [40, 41]. The oblimin rotation, an oblique rotation method, was used to allow the loadings to assort naturally without imposing orthagonality. Drug resistances with component loadings that had an absolute value greater than 0.4 were considered to be important and were assigned to the respective rotated component [42]. The rotated components were compared to the respective R-nets to evaluate agreement between the two methods.

## Results

### Regularization penalty selection

**Fig 1:**
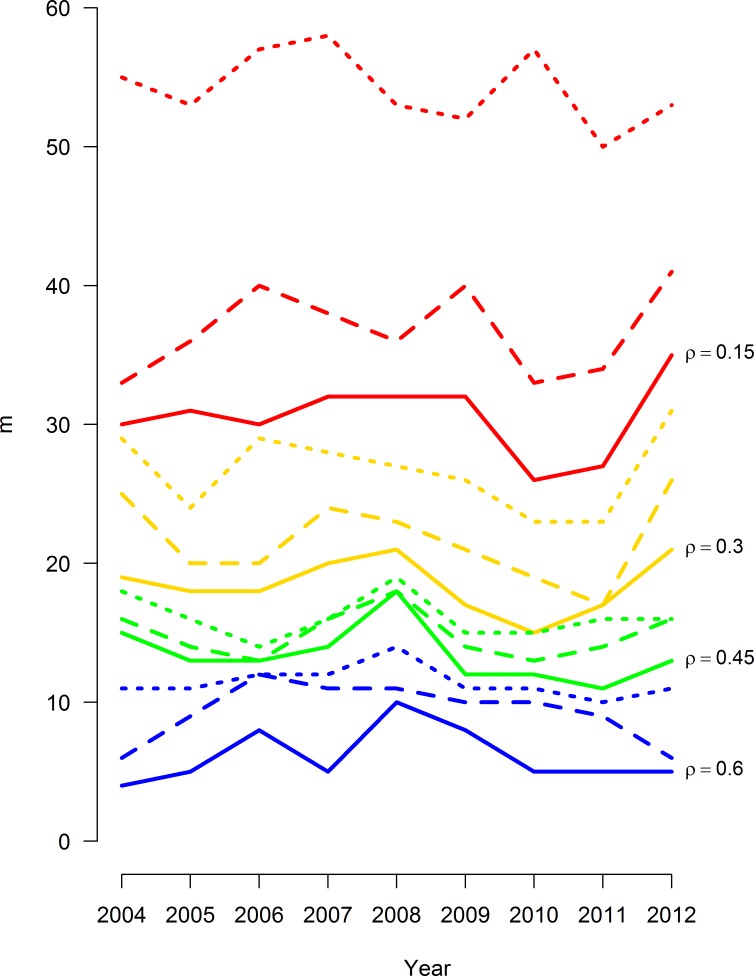
Size of Markov networks of AMR surveillance data over time, stratified by L_1_ regularization value. Networks were generated via the graphical least absolute shrinkage and selection operator with 12 L_1_ penalty values (*ρ*) from 0.05 to 0.60. Antimicrobial data includes 16 antimicrobial drugs results from 14,418 *E*. *coli* isolates collected from chicken carcass rinsates and commercial chicken breasts between 2004 and 2012.

**Fig 2:**
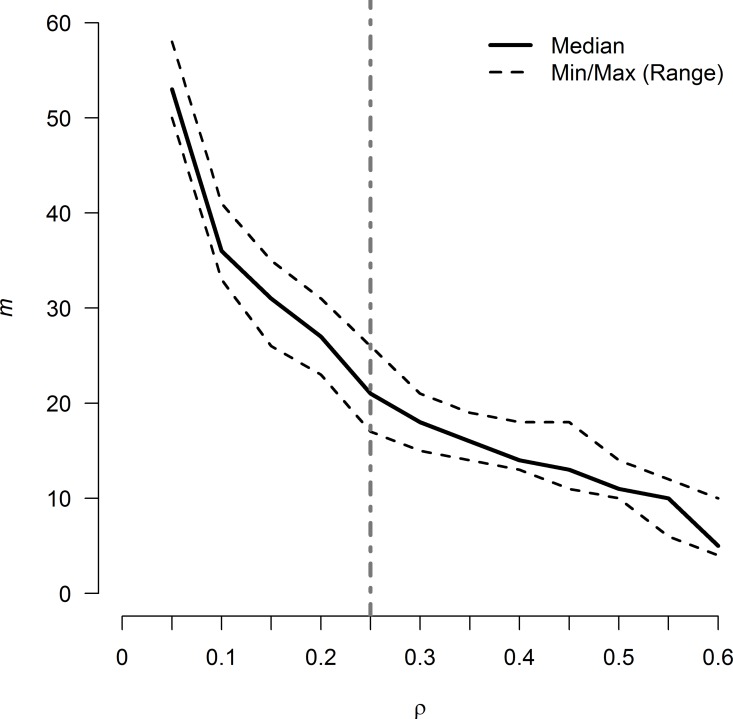
Size of Markov networks of AMR surveillance data (*m*) over L_1_ regularization values. R-nets were generated using 12 L_1_ penalty values (*ρ*), from 0.05 to 0.60 in increments of 0.05 for all 9 time periods in the study. The solid line represents the median graph size (*m*_50_) for the set *R*, which contains one R-net for each of the 9 years in the study at each evaluated value of *ρ*, and the dashed lines above and below *m*_50_ represent the maximum and minimum size of *R*, respectively. R-net size decreased rapidly until *ρ* ~ 0.3, where the slope started to stabilize. The slope of *m*_50_ appeared to change most abruptly near *ρ* = 0.10 and *ρ* = 0.25. The vertical line at *ρ* = 0.25 represents the set of R-nets presented in Fig 3. The set of R-nets generated at *ρ* = 0.10 are presented in S3-1 Fig with further discussion in S3.

**Fig 3:**
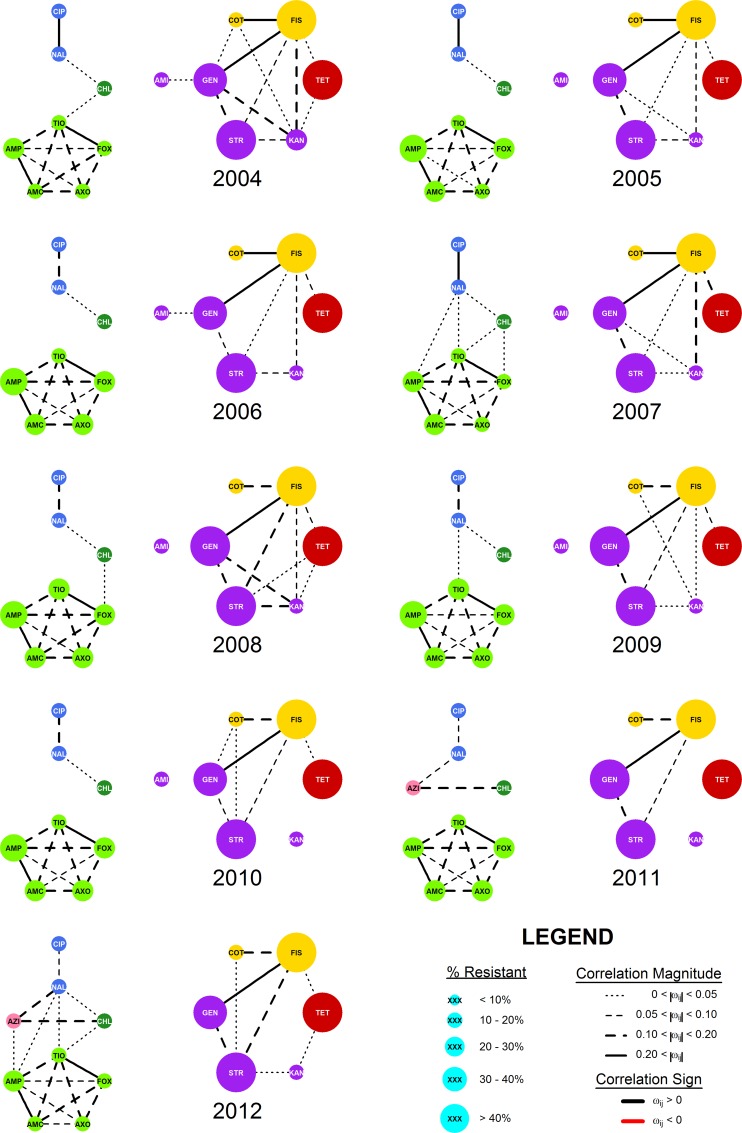
Markov networks of AMR surveillance data. Nine weighted Markov networks representing AMR data consisting of 14,418 total isolates of *E*. *coli* collected from chicken carcass rinsates and commercial chicken breasts from each year from 2004 to 2012. Networks were generated via the graphical least absolute shrinkage and selection operator with *ρ* = 0.25. The edges were decorated with line weights and styles to indicate the relative |*ω*_*ij*_| magnitude. Vertex size indicates the percent of isolates with an MIC meeting or exceeding the published breakpoint for respective drug (Table 2). Vertex colors indicate the Class of drug associated resistance as follows: Vertex colors indicate the Class of drug associated resistance as follows: β-lactams–light green, quinolones–blue, aminoglycosides–purple, sulfonamides–yellow; chloramphenicol–dark green; tetracycline–red; macrolide–pink.

The range of *ρ* evaluated was fully contained in the useful range identified above, with 0 < |*ϑ*_*ij*_| < 2.7 for all years in the study. The upper region of the possible penalties (0.6 < *ρ* < 2.7) was not evaluated since the networks generated in this region would have produced networks too sparse to be informative. The density of R-nets over all *ρ* values and years ranged from 4.8% to 57.1% over the evaluated range of *ρ*, and 0.3 ≤ *ρ* ≤ 0.5 generated graphs with very similar densities between 10% and 20%, indicating consistent graph structures in this range of *ρ* (Fig 1) and changes in the slope of ${\bar{m}}_{50}$ over *ρ* were noted at *ρ* = 0.10 and *ρ* = 0.25 (Fig 2). In general, the R-nets generated by *ρ* = 0.10 were too dense to easily interpret and several unstable cycles were noted in *R’*_2008_, *R’*_2009_ and *R’*_2010_. The R-nets generated by *ρ* = 0.25 were sparse enough to be interpreted with reasonable effort. Additionally, *R’* under *ρ* = 0.25 contained no unstable cycles since all partial correlations were positive. The latter penalty of *ρ* = 0.25 was selected as the common penalty and used to generate *R* and *R’* interpreted below (Fig 3). The supplemental material provides an overview of *ρ*’s impact on density and modularity (S2 Text) and a more in-depth description of *R* and *R’* conditioned on *ρ* = 0.10 (S3 Text).

### Network structure

**Fig 4:**
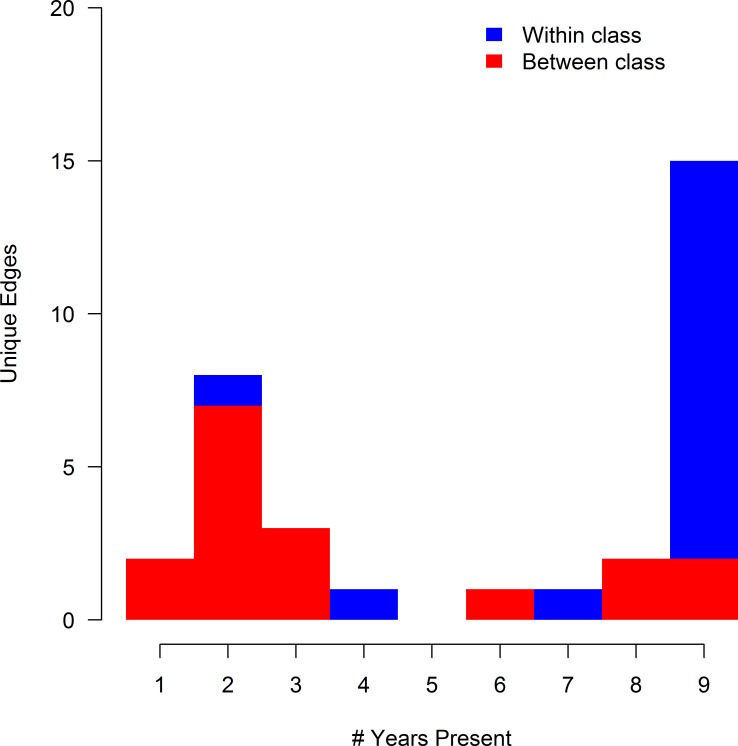
Histogram of edge frequency in Markov networks of AMR surveillance data. Thirty-three unique edges were identified in 9 Markov networks estimated using AMR data from 14,418 total isolates of *E*. *coli* collected from chicken carcass rinsates (USDA-ARS) and commercial chicken breasts (FDA) between 2004 and 2012. Networks were generated via the graphical least absolute shrinkage and selection operator with *ρ* = 0.25. Edges are categorized by the types of antibiotic resistances they joined, either resistances from the same class (blue) or different classes (red).

**Fig 5:**
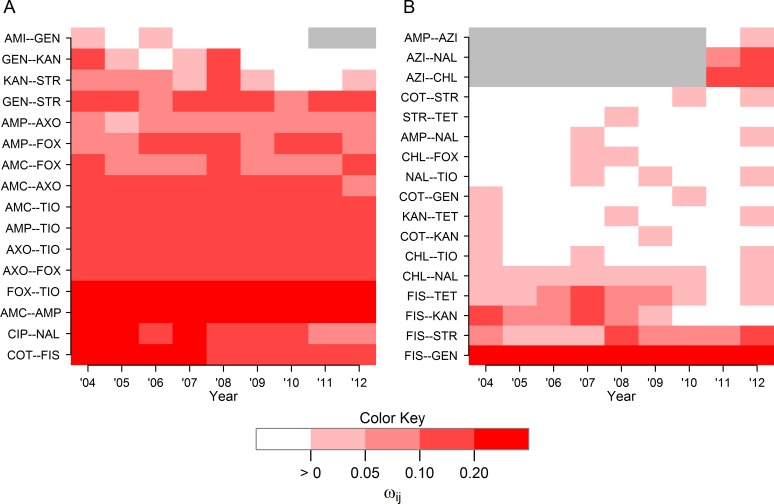
Heatmap of non-zero partial correlations between drug resistances over time. (A) Edge weights for 16 edges between resistances of drugs of the same class and (B) 17 edges between resistances to drugs of different classes in Markov networks of AMR data. Resistance data was collected from 14,418 total isolates of *E*. *coli* collected from chicken carcass rinsates and commercial chicken breasts between 2004 to 2012. Networks were generated via the graphical least absolute shrinkage and selection operator with *ρ* = 0.25. Grey areas represent years of missing data due one of the antibiotic drugs was not included in the panel (AMI after 2010 and AZI prior to 2011).

A total of 119 unique edges could have observed during the entire study period (the AZI-AMI edge could not have been observed since both drugs were never included in the same year), and 105 edges could have been observed in each year. Of these 119 unique edges,33 unique edges were observed in at least one year of the study, and 15 appeared in all 9 years. A bimodal distribution was noted in the frequency of edge appearance, with one group of edges observed in 1 to 4 years, and another group of edges observed for 6 years or more (Fig 4). Sixteen of the 33 observed edges found represented resistance relationships between drugs of the same class (blue areas of Fig 4), most of which were present in all nine years during the study (13/16 within-class edges) indicating the relative stability of these relationships during the study period (Fig 5A). Seventeen between-class edges (red areas of Fig 4) were identified, of which only 4 were present for 8 or 9 years. The majority of between-class edges (12/17 between-class edges) were present for 3 years or less (Fig 5B).

### Graphical parameters

No significant trend in $\bar{m}$ over time was noted (Spearman’s rho = -0.14, p = 0.71). Two dense subgraphs of R were noted to be frequently present. The first frequent dense subgraph *R*_BLA_ contained the five β-lactam drug resistances AMC, AMP, AXO, FOX, and TIO and formed a clique in every *R*_*y*_. The second frequent dense subgraph *R*_AST_ included GEN, KAN, STR, COT, FIS and TET, and ${\bar{m}}_{\mathrm{AST}}$ exceeded 50% in six out of the nine years.

Individual vertices demonstrated several different patterns of degrees during the study period, and *d* ≤ 6 for all vertices and time periods. For the β-lactam drugs, *d* = 4 was typical but occasionally increased to 5 or 6. Different patterns were noted among the degrees of the aminoglycosides: *d*_KAN_ was high the earlier in the study and decreased in later years (3 ≥ *d*_KAN_ in 2009 and prior and 2 ≤ *d*_KAN_ in 2010 and later), *d*_GEN_ and *d*_AMI_ were more consistent, and *d*_STR_ varied widely and without a clear pattern. In every time period, *d*_CIP_ = 1, corresponding to its edge with NAL, but *d*_NAL_ tended to increase through the study. The values for *d*_CHL_, *d*_FIS_, *d*_COT_, and *d*_AZI_ changed little during the study period. There was substantial variation in *d*_TET_ and did not appear to follow a pattern.

**Fig 6:**
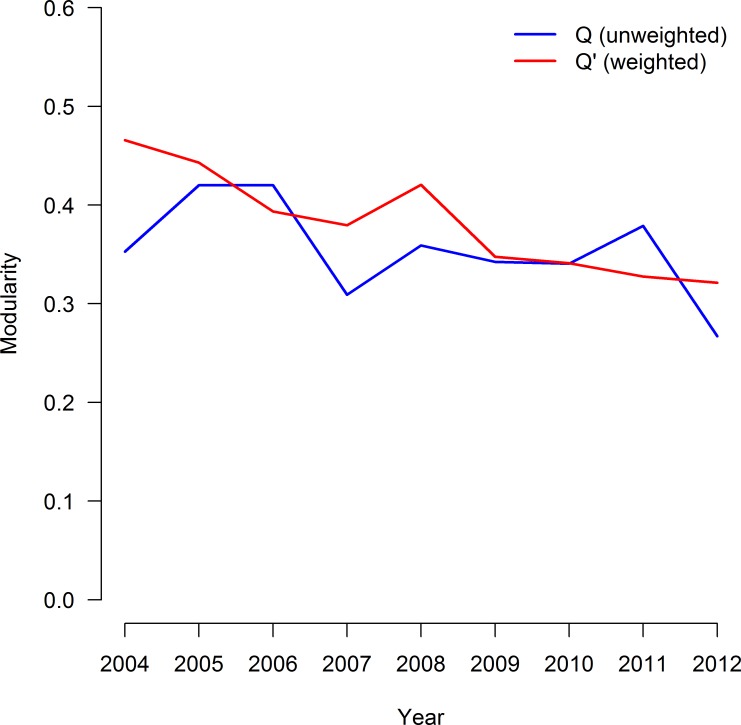
Modularity in Markov networks of AMR surveillance data over time. The unweighted modularity (*Q*, blue line) and weighted modularity (*Q'*, red line) over time for Markov networks of AMR data based on the class (See Table 1). The data set consisted 14,418 total isolates of *E*. *coli* collected from chicken carcass rinsates and commercial chicken breasts from each year from 2004 and 2012. Networks were generated via the graphical least absolute shrinkage and selection operator with *ρ* = 0.25. A statistically negative trend over time was noted in *Q’* (p < 0.005), but not in *Q* (p = 0.18).

Modularity defined by class in the unweighted R-nets ranged from *Q*_2012_ = 0.267 to *Q*_2005_ = *Q*_2006_ = 0.420. Modularity was not significantly associated with time (Spearman's rho = -0.49, p = 0.18; Fig 6). When weighted by partial correlation, modularity estimates ranged from *Q'*_2012_ = 0.321 to *Q'*_2005_ = 0.466, and a statistically-significant decreasing trend was noted in *Q'* (Spearman's rho = -0.95, p < 0.005).

### Principal component analysis

**Fig 7:**
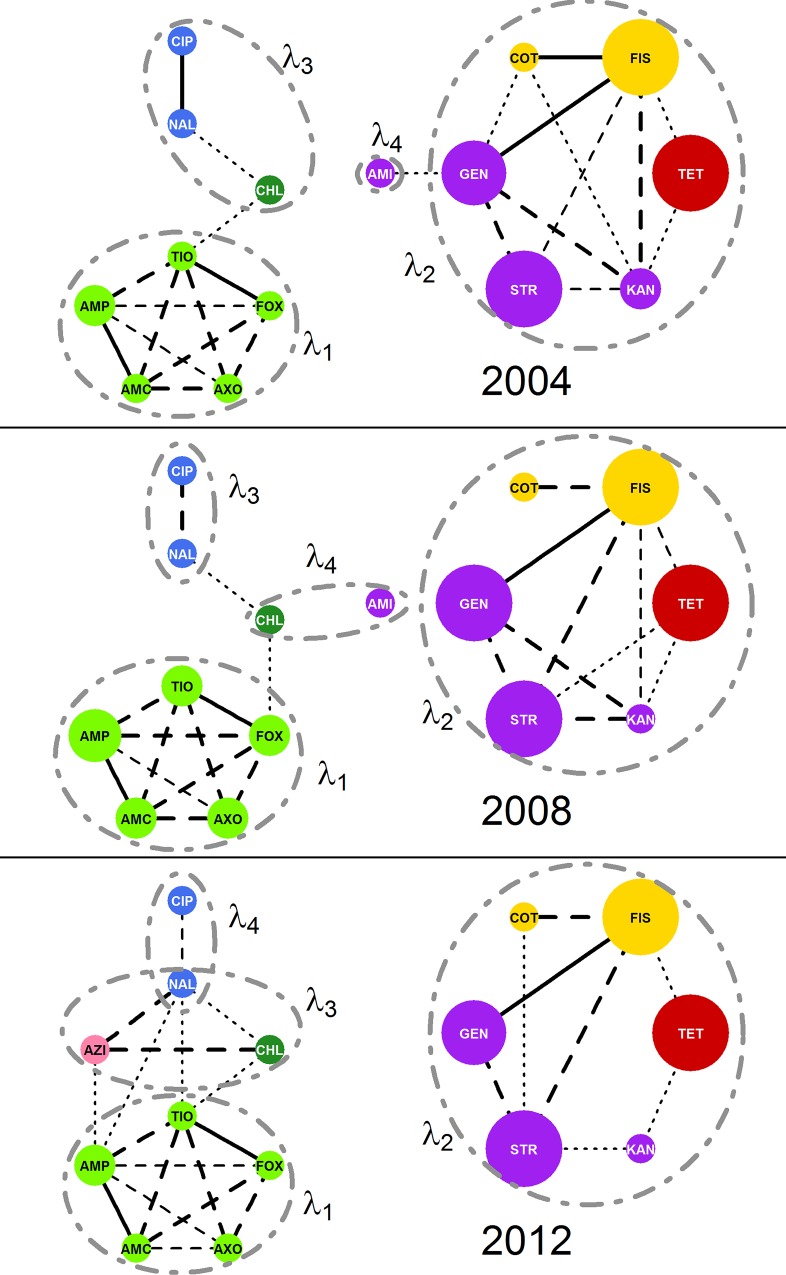
Markov network structure versus oblimin-rotated components. Comparison of Markov network structures (*R'*_*y*_) and oblimin-rotated components (PCA) show good alignment indicating a both methods give similar result. Networks and components were estimated from *E*. *coli* isolates from chicken carcass rinsates and commercial chicken breasts collected in 2004 (*n*_2004_ = 2097), 2008 (*n*_2008_ = 1292; B), and 2012 (*n*_2012_ = 1376). Networks were generated via the graphical least absolute shrinkage and selection operator with *ρ* = 0.25. Sets of variables with loadings > 0.4 on each component are circumscribed with dashed circles and labeled with the component's eigenvalue (*λ*).

Principal component analysis was performed on **Σ**_2004_, **Σ**_2008_, **Σ**_2012_. In each of the three years, four components were identified for extraction and oblimin rotation. Combined, these rotated components accounted for 61%, 64%, and 61% of the overall variance in log_2_MIC in 2004, 2008, and 2012 respectively. The loadings for each component were similar, though not identical, over all three years (Table 3). Correlations between the rotated components were small, with -0.10 ≤ r ≤ 0.25. Most rotated components aligned with subgraphs with $\bar{m}$ > 50% (Fig 7).

**Table 3:** Eigenvalues (λ) and loadings for the oblimin-rotated components identified in the principal component analysis (PCA) of the Spearman's rank correlation matrix of MICs to 15 drugs from the NARMS study in 2004 (*n*_2004_ = 2097), 2008 (*n*_2008_ = 1292), and 2012 (*n*_2012_ = 1376). Variables with loadings > 0.4 were used to interpret the component, and the density of the induced subgraphs containing the variables with loading > 0.4 for each component are provided in ${\bar{m}}_{\lambda }$. The ${\bar{m}}_{\lambda }$ of components consisting of a single variable were undefined (N/D). Resistance results for azithromycin (AZI) was not available in 2004 and 2008, and resistance results for amikacin (AMI) was not available in 2012.

	2004				2008				2012			
	*λ*_1_	*λ*_2_	*λ*_3_	*λ*_4_	*λ*_1_	*λ*_2_	*λ*_3_	*λ*_4_	*λ*_1_	*λ*_2_	*λ*_3_	*λ*_4_
* *	*3*.*31*	*2*.*94*	*1*.*70*	*1*.*20*	*3*.*71*	*3*.*10*	*1*.*57*	*1*.*29*	*3*.*41*	*2*.*72*	*1*.*78*	*1*.*21*
AMC	0.85				0.89				0.83			
AMP	0.81				0.85				0.81			
AXO	0.77				0.84				0.82			
FOX	0.79				0.83				0.79			
TIO	0.81				0.85				0.81			
AMI				0.91				0.81	*-*	*-*	*-*	*-*
GEN		0.73				0.83				0.76		
KAN		0.67				0.69				0.47		
STR		0.63				0.74				0.75		
CIP			0.78				0.87					0.94
NAL			0.85				0.80				0.55	0.49
FIS		0.88				0.87				0.85		
COT		0.71				0.59				0.65		
TET		0.49				0.5				0.46		
CHL			0.49					0.61			0.75	
AZI	*-*	*-*	*-*	*-*	*-*	*-*	*-*	*-*			0.82	
${\bar{m}}_{\lambda }$ (%)	100	73.3	66.7	N/D	100	66.7	100	0	100	53.3	100	100

## Discussion

The representation of AMR surveillance data using Markov networks generated via the graphical LASSO is a novel method to characterize potential collateral resistances in bacterial populations. The graphical nature of this method lends itself to simple visualization which allows complex relationships to be communicated clearly. The structures of *R*_2004_, *R*_2008_, and *R*_2012_ are similar to the variance structures identified by the respective PCA results, but the R-nets provide results that are simultaneously more detailed and more interpretable than the results from PCA.

Several resistance relationship patterns appeared in the models over time. Resistances to the β-lactam drugs were consistently and strongly related to each other in all years, generating the complete induced subgraph *R*_BLA_. These patterns of related classes are likely an example of cross-resistance, though pleiotrophic and co-resistance mechanisms may also be present. The elements of *R*_AST_*(V)* did not represent resistances to a single class of drugs or even drugs targeting a common metabolic pathway. A similar grouping of resistances without GEN was previously described in beef cattle, where it was attributed to antibiotics frequently used in production medicine [23]. The quinolone drugs were always correlated with each other, and while CIP was only correlated with NAL, NAL was additionally correlated with CHL, TIO, and AMP in some years. Additionally, these patterns are consistent with those seen in the PCA of the same data, and similar to patterns seen in a previous study of AMR relationships in *E*. *coli* [23].

Evaluation of the graphical parameters provided additional insight into changes in the joint distributions of resistance over time. No temporal trend was apparent in $\bar{m}$ indicating that, on average, the amount of interconnectivity of AMR in this population of *E*. *coli* did not change substantially over time. The negative trend noted in *Q'* indicates a shift towards stronger relationships between drugs of different classes, weaker relationships between drugs of the same class, or both. Visual evaluation of Fig 5A and 5B suggests both processes may be occurring, with within-class edges becoming slightly weaker over time and the appearance of more, stronger between-class edges. Lower weighted modularity values over time were consistent across the common penalties and may indicate a concerning increase in co-resistance and pleiomorphic mutations in this population of *E*. *coli*. Neither the source of the decreasing trend in *Q’* or the behavior of any specific edge can be assigned without isolate covariate data, which were not available for data from the NARMS study. Quantification of specific network structures into simple numeric criteria is one of the major advantages of graphical models. While some information about a graph’s structure is lost when the structure is condensed into a simple criterion, these criteria greatly facilitate the comparison of graphs. Parallel interpretation of multiple criteria, as is done here with *Q*, *Q’*, and $\bar{m}$, can provide a more complete description.

It should be emphasized that the R-nets describe the joint relationships of resistances at the population level, hence little can be inferred about the genotype or the phenotypic resistance profile of an individual isolate from these results. At best, a probabilistic statement can be made about some MIC values given knowledge about the others. It is also not currently possible to infer the source of the resistance or resistance relationships based on these models. The edges of the R-nets represent potential resistance relationships, but existence of the edge alone is not sufficient to induce collateral resistance. For example, even though GEN and FIS were adjacent, GEN will not affect FIS unless there exists a concurrent selection pressure for or against gentamicin resistance. Induction of collateral resistance for FIS by GEN requires the combination of the FIS-GEN resistance relationship and the selection for GEN, potentially from the therapeutic use of gentamicin. This illustrates that knowledge of the resistance relationships alone is insufficient to determine how the R-nets have influenced the patterns of resistance observed; antimicrobial use data are also needed and combining R-net results with exposure data is a topic of ongoing research. Despite this limitation, the R-nets can still identify phenotypic resistances of interest prospectively, specifically vertices of relatively high degree.

Application of the methods to multiple strains from varied sources will allow the opportunity to objectively identify resistance patterns, and potentially enable predictions about resistance relationship evolution based on previously observed R-nets. There are several applications where the population-level focus of the currently described method would be useful. First, this method could be directly applied to AMR surveillance in health care facilities where local evolution of highly resistant bacterial strains is a major concern [7]. While the dataset used to demonstrate the current method was relatively large and *n* >> *m* even in the unpenalized case, the graphical LASSO can perform well even when the number of parameters to estimate exceeds the number of available observations [43]. Therefore, the current method may be employed to estimate *R* even when the sample size is modest, as may be the case in a single health care facility. R-nets can be estimated retrospectively from clinical data to monitor for antimicrobials with a high degree, indicating a high risk of extensive collateral resistance. If drug use data were available from patient records, limited inference could be made about how an MDR strain evolved in a clinical environment. If appropriate negative correlations were noted, a selection inversion could also be attempted [21]. The R-nets could also be applied to monitor emergence of novel resistance relationships at any scale over time. Knowledge of resistance relationship dynamics will help improve clinical decision making by informing the physician about what resistances may altered by use of a specific drug. The R-nets can help inform policy making by similarly tracking resistance relationships and detecting resistance of relationships of concern, and can help facilitate AMR research by screening large numbers correlations to locate non-trivial associations.

The R-nets revealed several interesting patterns of AMR involving the resistances the quinolones drugs NAL and CIP. In *E*. *coli*, quinolone resistance may be increased by mutations in the A and B subunits of DNA gyrase [9, 44] and mutations in *parC* and *parE*, which encode subunits of DNA topoisomerase IV[45–47]. Increases in NAL and CIP due to these mutations would be expected to lead to a strong relationship between NAL and CIP, but no other drugs in the panel since the quinolones are the only class of antimicrobials affected by DNA gyrase and topoisomerase mutations. This relationship was observed, with *ω*_NAL,CIP_ > 0.1 in most years indicating a consistent strong NAL-CIP edge. Increased expression of active, non-specific efflux pumps in *E*. *coli* is an alternate and complementary mechanism of quinolone resistance, and also increases resistance to chloramphenicol, tetracycline, and other classes of drugs [10, 11, 48]. If efflux pumps were an important mechanism of quinolone resistance in the sampled population of *E*. *coli*, correlations should be noted among NAL, CIP, TET and CHL and a dense subgraph including these vertices should be noted. However, no such subgraph was noted: TET was never found to be correlated with CIP, NAL, or CHL, and *ω*_CHL,NAL_ < 0.05 most years. Efflux pump expression may explain the weak CHL-NAL edge but, overall, these results would appear to support the conclusion that efflux pumps expression was not an important source quinolone resistance, or at least was not a major contributor to resistance in this population.

Given *ρ* = 0.25, every edge in every year represented a positive partial correlation in resistances, indicating that, on average, increasing resistances to one drug was only associated with increased, and never decreased, resistances of the adjacent vertices. Among the unpenalized partial correlations, positive partial correlations outnumbered negative partial correlations by about a 2-to-1 margin, and the median absolute value of negative partial correlations was about half that of positive correlations. Negative partial correlations were only noted when ρ ≤ 0.15. These findings are consistent with the phenomenon of genetic capitalism where the progeny of bacteria with at least one advantageous mutation tend to acquire other additional advantageous traits over time via recombination and HGT [6]. The relative weakness and infrequency of negative partial correlations compared to positive partial correlations is also consistent with the patterns seen in the resistance relationship networks of previous studies [22, 49]. One application of R-nets and similar networks is to identify pairs or larger groups of collaterally susceptible antibiotics to create a selection inversion: a reduction in overall AMR via strategic antibiotic use [21, 22]. Without any negative partial correlations, it is unlikely that a selection inversion could be achieved in this population of *E*. *coli*, but could be feasible in other populations.

The purpose of the L_1_ penalization in the graphical LASSO procedure is to eliminate trivial edges from the graph by reducing their corresponding elements of **Ω** to zero, but the penalization also biases the non-trivial elements towards zero, as well [27, 50]. This is effect is the ‘shrinkage’ aspect of the least absolute shrinkage and selection operator. For example, the estimated unpenalized partial correlation between AMC and AMP in 2012 is 0.62, but when estimated via the graphical LASSO with ρ = 0.25 this estimated partial correlation is reduced to 0.36; the latter estimate of ω_AMC, AMP_ has been moved towards 0, or shrunken, compared to the former due to the L_1_ penalization. This bias caused by the penalization should be kept in mind when interpreting the magnitudes of the edge weights since edges attributed to small penalized partial correlations may actually correspond to substantially larger partial correlations in the unpenalized matrix. The penalized partial correlations are still useful, though, because they provide a useful method to compare the relative strengths edges within a graph or graph series. Though *R(E)* = *R'(E)*, the unweighted models are not affected by this bias since **A** does not incorporate magnitude of the partial correlations. Hence, R may be preferable to R' for some applications.

One important limitation of Markov networks is that they cannot specifically identify the higher order dependence structure of joint distributions complete induced subgraphs [31]. Without additional information about the population or the random variables comprising by *R(V)*, the researcher must rely on induction to determine which covariance structure is more likely to be correct. For example, a complete induced subgraph of a Markov network with *k* = 4 could be the result of six separate pair-wise correlations, a combination of one 3-way and 3 pair-wise correlations, or a single 4-way correlation. The frequent dense subgraph *R*_BLA_ is a case in the current study where a higher order dependence structure may exist but cannot be specified. Due to the strength of the edges, consistency of edges over time, and similarity of the drugs in *R*_BLA_, it would be reasonable to attribute the observed structure to a single 5-way correlation element, but combinations of multiple lower order interactions are possible.

The R-nets are unable to distinguish the biochemical or genetic mechanisms responsible for the observed phenotypic relationships, and individual edges may represent multiple categories of collateral resistance. However, some inferences may be reached via induction based our biological knowledge of the resistance mechanisms. Edges between drug resistances in the same class could be caused by cross-resistance mechanisms, or pleiotropic mutations affecting the common site of multiple drugs. For example, it is likely that β-lactamase enzymes, variations in penicillin-binding proteins or both are responsible for the dense subgraph of AMP, AMC, AXO, FOX, and TIO[51, 52]. Edges between drug resistances that belong to different classes could be caused by either co-resistance or pleiotropic resistance separately. Multiple different mechanisms may be present active in a single R-net, and multiple types of collateral resistance may contribute to a particular edge. The AZI-CHL edge was observed both years where AZI ∈ *R*_*y*_*(V)*, and both drugs target the 50S ribosomal subunit. This relationship could be attributed to a pleiotropic mutation of the 50S subunit affecting the action of both AZI and CHL, co-resistance from genes residing on a plasmid or in the same genetic cassette, or both. Genotypic data could be used to elucidate the mechanisms underlying the observed phenotypic correlations, and the structure of R may provide insight into the genetic mechanisms leading the MDR bacteria. An important step in the validation of the current method will be to demonstrate the structure of *R* generated by a bacterial population conforms to the combinations of resistance genes present in the sampled bacteria.

The selection of *ρ* in the current method is of particular importance due to its influence on the generated networks. Here, *ρ* = 0.25 was selected because this value produced biologically coherent graphs in an informative range of densities around 20%. Larger or smaller values of *ρ*, such as the scenario presented in S3, may be appropriate for other applications. The subjectivity of the selection is mitigated since **Θ** is uniformly penalized, so any edges that are trivial at a given value of *ρ* will also be trivial at all higher values. Future work will explore additional methods for selecting *ρ* to improve standardization across studies.

The graphical nature of this method lends itself to simple visualization, allowing for complex relationships to be communicated clearly and additionally provides a framework for further analysis where the presence and magnitude of the partial correlations provide an outcome for evaluation via other statistical methods. Continuous covariates, including the number of gene copies present in an isolate, could be included as nodes in an R-net in addition to the drug resistances. Edges between a covariate node and resistance nodes could identify relationships similar to collateral resistance. Covariates of high degree may identify avenues for the indirect induction of AMR similar to collateral resistance, albeit indirectly. Graphical parameters for these covariate nodes such as centrality could provide additional insight into the evolution of highly resistant strains. A separate strategy for comparing R-nets is to test for differences in **Ω** generated by bacterial subpopulations. A number of methods for comparison of covariance matrices have been proposed [53] and may present a feasible proxy method to compare *R’* structure since *R’* is defined by **Ω**. Samples can be stratified using isolate date, e.g., isolate source, time interval of collection, genotype, etc., *R’* estimated for each stratum, and **Ω** compared across the strata. Unlike the strategies for comparison of covariates described earlier, which included covariates as nodes *within* the network, **Ω** comparisons estimate separate R-nets based on covariates *external* to the network and therefore must identify covariates of interest *a priori*. Additional work is needed to determine how to best evaluate covariates in R-nets, and it will be important to validate the phenotypic findings against genetic data to improve the interpretability of results.

We chose evaluate joint distributions of log-transformed MIC values, but it is common practice to dichotomize MIC values into susceptible or resistant categories based on breakpoints when analyzing resistance data [14, 49, 54]. The transformed AMR data contain more information than dichotomized results, and therefore analyses of the continuous data is more powerful than similar analyses of dichotomized data [55, 56]. The dichotomization of MIC values is also dependent on the selected breakpoints, which are based on clinically-relevant drug concentrations, and not based on the distribution of MICs within bacterial populations [57]. Breakpoints may also vary over time and geographic region [57–61]. We believe the networks developed from continuous resistance data more accurately represent the resistance relationships than networks based on more traditional dichotomized results.

Previous studies have used directed graphical models to describe the structure joint distributions of resistance in *E*. *coli* [22, 49, 62]. Directional edges represent types of causal relationships in the system they represent. In some cases, it is appropriate to assign directionality to AMR relationships, e.g., collateral resistance generated by *in vitro* selection for resistance to individual drugs [22] or the association between genes and phenotypical resistances [62]. In many other situations, including observational or surveillance studies of AMR where antimicrobial use is unknown, causality of an AMR relationship, if any exists, must be assumed [49]. In contrast, Markov networks are undirected and have no implied causality, which avoids the risk of incorrect assumptions regarding cause and effect.

While effective for describing and visualizing AMR relationships, the R-nets cannot provide information about univariate changes to the MICs over time. Hence, this method is intended complement current surveillance methodologies, not replace them. It was noted that the majority of MICs test ranges included at most 1 dilution above the CLSI suggested breakpoint for resistance, and some MIC test ranges had as few as two dilutions. Increasing the number of dilutions tested for MICs would capture more information and increase the accuracy of the R-nets.

## Conclusions

The graphical models presented provide a novel method of mapping resistance relationships in observed in AMR surveillance data. The R-nets present a powerful and useful tool can provide insight into the evolution of MDR bacterial strains and allow simple visualization of complex AMR data. Future work is needed to validate the R-nets against genotypic results to confirm the observed phenotypic resistances accurately represent the underlying biochemical mechanisms of AMR. The application of the method to pathogenic species of bacteria, including MRSA, *Salmonella* spp. and *Campylobacter* spp., is planned and may provide insight into antimicrobials driving the evolution and emergence MDR bacteria.

## Supporting Information

S1 Text Data source and sample sizes. Summary of *E*. *coli* isolates and resistance testing methods for NARMS data used to demonstrate the R-net method. Includes table S1-1 and S2-2.
(DOCX)

S2 Text Exploration of results under various L_1_ penalties. An expanded evaluation of how the L_1_ penalty effects the structure of R-nets. Includes tables S2-1 and S2-2.
(DOCX)

S3 Text Alternate R-net Results. A review of the structure of the *E*. *coli* R-nets conditioned on L_1_ = 0.10. This penalty was identified using the objective methods described in S2, but was rejected in favor of a higher penalty due to difficulties in interpreting the dense networks produced here. Includes tables S3-1 and S3-2.
(DOCX)

S1 Fig Sample size for *E*. *coli* by NARMS agency and year. A stacked histogram representing the 14,418 *E*. *coli* isolates provided to the NARMS study between 2004 and 2012 by the United States Department of Agriculture Agricultural Research Service (USDA-ARS, red portions) and Food and Drug Administration (FDA, blue portions) from chicken carcass rinsates and commercially-packed chicken breast products, respectively.
(TIF)

S2 Fig Comparison of proportion of within- and between-class edges under multiple L_1_ penalties. Edges joining resistances to drugs of a similar class (‘within-class’ edges) were substantially more robust to low regularization penalties (ρ < 0.30), while edges joining resistances of different antimicrobial classes were disproportionately removed by the lower penalties. The difference in behavior between these two types of edges led to differences in unweighted modularity based on the selected penalty. The drug resistances were grouped into one of the following classes based on drug structure: aminoglycosides, β-lactams, fluoroquinolones (including NAL), sulfonamides, tetracyclines, and macrolides (see Table 1). Network structures were estimated from MIC data for 16 drugs from 14,418 E. coli isolates collected by the FDA and USDA during 2004–12. The vertical line at ρ = 0.25 indicates the penalty used to generate R and R’ in the presented study.
(TIF)

S3 Fig Markov networks of AMR surveillance data under an alternate regularization penalty. Nine weighted Markov networks representing AMR data consisting of 14,418 total isolates of E. coli collected from chicken carcass rinsates and commercial chicken breasts from each year from 2004 to 2012. Networks were generated via the graphical least absolute shrinkage and selection operator with ρ = 0.10. In general, these graphs were considered too dense to be informative and included unstable cycles in several instances. The edges were decorated with line weights and styles to indicate the relative |ωij| magnitude and the sign of the partial correlation. Vertex size indicates the percent of isolates with an MIC meeting or exceeding the published breakpoint for respective drug (See Table 2). Vertex colors indicate the Class of drug associated resistance as follows: β-lactams–light green, fluoroquinolones–blue, aminoglycosides–purple, sulfonamides–yellow; chloramphenicol–dark green; tetracycline–red; macrolide–pink.
(TIF)

S4 Fig Histogram of edge frequency in Markov networks of AMR surveillance data under an alternate regularization penalty. Sixty-one unique edges were identified across 9 Markov networks estimated using AMR data from 14,418 total isolates of E. coli collected from chicken carcass rinsates (USDA-ARS) and commercial chicken breasts (FDA) between 2004 and 2012. Networks were generated via the graphical least absolute shrinkage and selection operator (LASSO) with ρ = 0.10. A similar bimodal distribution to that seen under ρ = 0.25 was noted, but with a higher peak at the lower end of the plot representing infrequently present edges. Edges are categorized as either within-class (blue) or between-class (red) edges.
(TIF)

S5 Fig **Heatmap of non-zero partial correlations between drug resistances over time under an alternate regularization penalty** (A) Edge weights for 17 edges between resistances of drugs of the same class and (B) 44 edges between resistances to drugs of different classes in Markov networks of AMR data. Red and green coloring represent the magnitude of the positive and negative partial correlations, respectively, defining the edge. Grey areas represent years when one of the antibiotic drugs was not included in the panel (AMI after 2010 and AZI prior to 2011) and the edge could not be observed. The AMI-KAN edge was the only within-class edge found under ρ = 0.10 that did not appear under ρ = 0.25. The lower penalty found many more unique between-class edges (44 vs 17), but many of these were transient. Edges that appeared transiently when ρ = 0.25 were more consistently present under ρ = 0.10.
(TIF)

S6 Fig Modularity in Markov networks of AMR surveillance data over time. The unweighted modularity (Q, blue line) and weighted modularity (Q', red line) based on the class of drug associated with the resistance (See Table 1) for Markov networks of AMR data over time. Networks were generated via the graphical least absolute shrinkage and selection operator with ρ = 0.10. A statistically negative trend over time was noted in both Q (Spearman’s rho = -0.86, p < 0.005) and Q’ (Spearman’s rho = -0.96, p < 0.005).
(TIFF)
