# Supplementary material for: Markov Networks of Collateral Resistance: National Antimicrobial Resistance Monitoring System Surveillance Results from Escherichia coli Isolates, 2004-2012
Source: PLoS Comput Biol. 2016 Nov 16;12(11):e1005160. doi: 10.1371/journal.pcbi.1005160 (PMC5112851; doi:10.1371/journal.pcbi.1005160)
Supplement: S1 Text — Summary of E. coli isolates and resistance testing methods for NARMS data used to demonstrate the R-net method. Includes table S1-1 and S2-2. (DOCX) [file pcbi.1005160.s001.docx]

**Supplement #1. Data source and sample sizes.**

Data from the NARMS studies used to estimate the resistance relationship networks were limited to retail chicken meat and chicken carcass rinsates collected by the FDA and USDA, respectively. Only *E. coli* isolates were selected since this organism is classified as an indicator organism by the NARMS report and has only one species of interest. Data prior to 2004 was omitted from due to inconsistency of AMR panel drugs and data from 2013 was omitted due to the absence of chicken carcass results. The total number of isolates from each of the nine years ranged from 614 in 2011 to 2,232 in 2004. The data subset was restricted to FDA-sampled commercial chicken meat (*n*_FDA_ = 3,215 isolates) and ARS-sampled chicken carcasses (*n*_ARS_ = 11,203 isolates) because other meat types (beef, pork, and turkey) did not appear in the USDA data during the selected years. The number of isolates sampled by the USDA-ARS and FDA are shown in table S1-1, and relative sample sizes are provided in Figure S1.

Resistance results for 23 drugs were available for the selected *E. coli*. Some variation in the drug panels by agency and year was noted. The available results by drug and year are provided in table S1-2.

The MIC results for the following 16 drugs were included in the dataset: ampicillin (AMP), amoxicillin and clavulanic acid (AMC), ceftriaxone (AXO), cefoxitime (FOX), ceftiofur (TIO), amikacin (AMI), gentamicin (GEN), kanamycin (KAN), streptomycin (STR), nalidixic acid (NAL), ciprofloxacin (CIP), sulfisoxazole (FIS), trimethoprim and sulfamethoxazole (COT), chloramphenicol (CHL), tetracycline (TET), and azithromycin (AZI). Fourteen drugs appeared in the resistance panels every year, AMI was removed from panels after 2010, and AZI was added to panels in 2011. Hence, 15 drugs were included in each year’s panel (*k* = 15).

The remaining seven drugs, aztreonam (ATM), ceftazidime (CAZ), cefotaxime (CTX), cefepime (FEP), imipenem (IMI), piperacillin and tazobactam (PTZ), and sulfamethoxazole (SMX), had few resistance results and were excluded from our analysis. Six of these drugs (ATM, CAZ, CTX, FEP, IMI, PTZ) were excluded because MICs were only available for a very small proportion of isolates (< 20%) in the years they were included in the panel, and SMX was included in only one year during this period, and was therefore also excluded.

The MIC values were log_2_ transformed to express resistances as simple integer values. Isolates with specific MICs in excess of the maximum tested drug concentration were assigned log_2_MIC = log_2_MIC_max_ + 1 for the corresponding resistance. This adjustment was necessary to represent that isolates that grew at all tested drug concentrations (e.g., FOX > 2) were more resistant than isolates inhibited by the maximum tested drug concentration, but grew in all others (e.g., FOX ≤ 2). Isolates were classified as resistant or susceptible using drug breakpoints published by the USDA and the Clinical Laboratory Standards Institute (CLSI) for *Enterobacteriaceae* [1, 2].

REFERENCES

1. USDA-ARS. National Antimicrobial Resistance Monitoring System Animal Isolates [cited 2016 Mar 31]. Available from: <http://www.ars.usda.gov/Main/docs.htm?docid=6750&page=3>.

2. CLSI. Performance Standards for Antimicrobial Susceptibility Testing. Wayne, PA, USA: Clinical Laboratory and Standards Institute; 2016. Available from: <http://em100.edaptivedocs.com/>.

**Table S1-1.** Number of isolates of *E. coli* provided to the NARMS study between 2004 and 2012 by the United States Department of Agriculture Agricultural Research Service (USDA-ARS) and Food and Drug Administration (FDA) from chicken carcass rinsates and commercially-packed chicken breast products, respectively.

| Years | FDA | USDA-ARS | Total |
| --- | --- | --- | --- |
| 2004 | 400 | 1697 | 2097 |
| 2005 | 393 | 2232 | 2625 |
| 2006 | 418 | 1357 | 1775 |
| 2007 | 299 | 1510 | 1809 |
| 2008 | 306 | 986 | 1292 |
| 2009 | 315 | 876 | 1191 |
| 2010 | 357 | 941 | 1298 |
| 2011 | 341 | 614 | 955 |
| 2012 | 386 | 990 | 1376 |

**Table S1-2.** Number of MIC observations for 14,018 *E. coli* isolates for all 23 drugs in USDA-ARS and FDA AMR panels between 2004 and 2012.

| Year | AMC | AMI† | AMP | ATM* | AXO | AZI† | CAZ* | CHL | CIP | COT | CTX* | FEP* | FIS | FOX | GEN | IMI* | KAN | NAL | PTZ* | SMX* | STR | TET | TIO |
| --- | --- | --- | --- | --- | --- | --- | --- | --- | --- | --- | --- | --- | --- | --- | --- | --- | --- | --- | --- | --- | --- | --- | --- |
| 2004 | 3043 | 3043 | 3041 | 0 | 3043 | 0 | 0 | 3043 | 3043 | 3043 | 0 | 0 | 3043 | 3043 | 3043 | 0 | 3043 | 3043 | 0 | 0 | 3043 | 3043 | 3043 |
| 2005 | 3542 | 3542 | 3542 | 0 | 3542 | 0 | 0 | 3542 | 3542 | 3542 | 0 | 0 | 3542 | 3542 | 3542 | 0 | 3542 | 3542 | 0 | 0 | 3542 | 3542 | 3542 |
| 2006 | 2640 | 2640 | 2640 | 0 | 2640 | 0 | 0 | 2640 | 2640 | 2640 | 0 | 0 | 2640 | 2640 | 2640 | 0 | 2640 | 2640 | 0 | 0 | 2640 | 2640 | 2640 |
| 2007 | 2532 | 2532 | 2532 | 0 | 2532 | 0 | 0 | 2532 | 2532 | 2532 | 0 | 0 | 1022 | 2532 | 2532 | 0 | 2532 | 2532 | 0 | 1510 | 2532 | 2532 | 2532 |
| 2008 | 1988 | 1988 | 1988 | 0 | 1988 | 0 | 0 | 1988 | 1988 | 1988 | 0 | 0 | 1988 | 1988 | 1988 | 0 | 1988 | 1988 | 0 | 0 | 1988 | 1988 | 1988 |
| 2009 | 1891 | 1891 | 1887 | 102 | 1891 | 0 | 102 | 1891 | 1891 | 1891 | 102 | 101 | 1891 | 1891 | 1891 | 102 | 1891 | 1891 | 102 | 0 | 1890 | 1891 | 1891 |
| 2010 | 2119 | 2119 | 2119 | 116 | 2119 | 0 | 116 | 2119 | 2119 | 2119 | 116 | 117 | 2119 | 2119 | 2119 | 117 | 2119 | 2119 | 117 | 0 | 2119 | 2119 | 2119 |
| 2011 | 1684 | 0 | 1683 | 140 | 1684 | 1684 | 140 | 1684 | 1684 | 1684 | 140 | 140 | 1684 | 1684 | 1684 | 140 | 1684 | 1684 | 140 | 0 | 1684 | 1684 | 1684 |
| 2012 | 2199 | 0 | 2199 | 157 | 2199 | 2199 | 157 | 2199 | 2199 | 2199 | 157 | 157 | 2199 | 2199 | 2199 | 157 | 2199 | 2199 | 157 | 0 | 2199 | 2199 | 2199 |

*: Drug/resistance was excluded from all analyses because the drug was included in only year, or too few observations were present in all the years the drug was included in the panel


†: Drug/resistance was excluded from years where the drug was not included in the panel, but included in years where more complete data was available.
