# Supplementary material for: Markov Networks of Collateral Resistance: National Antimicrobial Resistance Monitoring System Surveillance Results from Escherichia coli Isolates, 2004-2012
Source: PLoS Comput Biol. 2016 Nov 16;12(11):e1005160. doi: 10.1371/journal.pcbi.1005160 (PMC5112851; doi:10.1371/journal.pcbi.1005160)
Supplement: S2 Text — An expanded evaluation of how the L1 penalty effects the structure of R-nets. Includes tables S2-1 and S2-2. (DOCX) [file pcbi.1005160.s002.docx]

**Supplement #2. Exploration of results under various L1 penalties.**

The LASSO’s L_1_ penalty parametrically determines the density and structure of sparse Markov networks inferred from correlation matrices. Since the general goal of the graphical LASSO is to estimate a data set’s true underlying multivariate covariance structure, selection of an appropriate penalty is important to reach accurate results. In general, the penalty should be high enough to remove trivial or unimportant edges, but not so high as to remove edges representing important relationships. An exploration of how various L_1_ penalty values impact the structure of the R-nets and their derived parameters is provided here to demonstrate how *ρ* = 0.25 was selected for further evaluation.

As *ρ* increased, the graphical density tends to decrease. Graphical density appeared to be stationary over time for all penalty values and no trend over time was apparent (Fig 1). Negative partial correlations were uncommon at all values of *ρ*. At *ρ* = 0.05, the lowest penalty evaluated, 26 of the 99 unique edges identified in at least one year ever represented negative partial correlations and only 78 (19%) edges were negative of all 410 edges identified in this set of R-nets. The negative edges are all but gone at *ρ* = 0.15,

Edges between resistances to drugs of the same class appeared to be much more robust to increasing penalties than did edges representing cross-class resistances (Fig S3). The cross-class edges are rapidly removed until *ρ* = 0.15, and don’t stabilize until *ρ* ≥ 0.30 where all but a few have been eliminated. In contrast, more than 80% of edges between drugs of a similar class are present until *ρ* > 0.30. This behavior is not particularly surprising since it could be expected for resistances to similar drugs to be highly correlated while resistances to unrelated drugs are typically weaker in comparison.

For all penalties evaluated, *Q* > 0 but there was substantial variation in its magnitude (Table S2-1). The changes in *Q* over *ρ* can be attributed to the different behaviors of the within- and between-class noted above. In general, when a graph is dense (*m* = *_k_*C_2_) or empty (*m* = 0), *Q* = 0. As *ρ* increases from 0 to 0.30, cross-class edges are lost more quickly than within-class edges and Q increases. After *Q* peaks at *ρ* = 0.30, then *Q* decreases as *ρ* increases because majority of remaining edges are within-class and are lost and as the networks become progressively sparser. Like *Q*, and empty graph has *Q’* = 0, but *Q’* is not necessarily 0 when a graph is dense because while all possible edges exist, they are not necessarily all of uniform weight. In the current data, *Q’* changes little in the range of 0.05 ≤ *ρ* ≤ 0.30, then steadily decreases above this range.

While *Q* and *Q’* are not necessarily robust over the penalties examined, the correlation between both forms of modularity and time were negative in nearly all cases (Table S2-2). The estimated Spearman’s correlations between *Q* and time was significant in six of the twelve evaluated penalty values, and p < 0.20 in most. The correlation of *Q’* and time was both strong and highly significant in all cases where *ρ* ≤ 0.50.

*Methods to select L_1_*

Several methods were attempted to select *ρ* objectively. Values of *ρ* from 0.01 to 0.60, incremented by 0.01, were evaluated to improve resolution compared to the set of twelve values of *ρ* used to evaluate the R-nets subjectively. The adaptive rho method for the graphical LASSO provides a hypothesis-driven test to determine a regime for the L_1_ penalty based on structure [1]. The graphs selected by this method varied by year, with some empty (*m_y_* = 0) and others having a single component (*m_y_* ≥ 30 in eight of nine years and *ρ* in the regime of 0.1 and 0.2). The adaptive rho method was not a useful tool for selecting *ρ* for the R-nets.

A method for selecting *ρ* using the model likelihood estimated by the glasso package (*L*) was proposed in the original study describing the method. In both the example case provided by Hastie, et al and the current study, *n* >> *m* even when *m̄* = 1 and *L* was maximized when *ρ* = 0. To encourage selection of a more parsimonious model, Akaike’s Information Criterion (AIC) was adapted to the log-likelihood results generated by the glasso package [2]. AIC penalizes the log-likelihood by the number of estimated parameters in the system, in this case *m_y_* (eq S1). The preferred model is that one which minimizes AIC.

| AIC*_y_* = 2*m_y_* – 2 ln (*L_y_*) | (Eq S1) |
| --- | --- |

Based on the year, the lowest values of AIC*_y_* were generated with the penalty on the range of 0.05 ≤ *ρ* ≤ 0.10, with 47% < *m̄_y_* < 56% and 31% < *m̄_y_* < 40% for the densest and sparsest cases, respectively. As discussed in the penalty selection section, a change in the slope of *m̄* over *ρ* was also noted at *ρ* = 0.10. In general, we found even the sparsest network generated by this method to be too dense to usefully interpret.

Upon seeing the results of these penalty selection methods, it was judged that a supervised, albeit subjective, method to select *ρ* would be superior. The set of R-nets generated by *ρ* = 0.10 is presented as an alternate case in the next supplementary section.

REFERENCES

1. Lockhart R, Taylor J, Tibshirani RJ, Tibshirani R. A significance test for the lasso. 2014:413-68. doi: 10.1214/13-AOS1175.

2. Akaike H. New Look at Statistical-Model Identification. Ieee T Automat Contr. 1974;Ac19(6):716-23. doi: Doi 10.1109/Tac.1974.1100705. PubMed PMID: WOS:A1974U921700011.

**Table S2-1**. Summary of unweighted (*Q*) and weighted (*Q’*) modularity estimates for *E. coli* R-nets from the 2004-2012 NARMS study. The data used to estimate network structure included MIC results for 16 drugs from 14,418 *E. coli* isolates collected by the FDA and USDA. Drug resistances were classified by the class of drug associated with resistances, and included aminoglycosides, amphenicols, β-lactams, fluoroquinolones, sulfonamides, tetracyclines, and macrolides (See Table 1). Mean (*Q̄*; *Q̄’*), median (*Q*_50_, *Q’*_50_) and range of modularity over the 9 years of the study are summarized based on the various L_1_ penalties (*ρ*) and were evaluated to determine a common penalty. All *Q* and *Q’* estimates were positive in all years.

|  |  | Unweighted | | |  | Weighted | | |
| --- | --- | --- | --- | --- | --- | --- | --- | --- |
| *ρ* |  | *Q̄* | *Q*_50_ | Range |  | *Q̄’* | *Q’*_50_ | Range |
| 0.05 |  | 0.084 | 0.089 | (0.048, 0.112) |  | 0.346 | 0.344 | (0.275, 0.411) |
| 0.10 |  | 0.180 | 0.167 | (0.112, 0.253) |  | 0.371 | 0.364 | (0.296, 0.441) |
| 0.15 |  | 0.247 | 0.245 | (0.162, 0.298) |  | 0.380 | 0.373 | (0.305, 0.446) |
| 0.20 |  | 0.278 | 0.290 | (0.200, 0.332) |  | 0.384 | 0.379 | (0.316, 0.455) |
| 0.25 |  | 0.354 | 0.353 | (0.267, 0.420) |  | 0.382 | 0.379 | (0.321, 0.466) |
| 0.30 |  | 0.391 | 0.400 | (0.298, 0.440) |  | 0.360 | 0.361 | (0.292, 0.458) |
| 0.35 |  | 0.379 | 0.382 | (0.322, 0.440) |  | 0.325 | 0.315 | (0.244, 0.441) |
| 0.40 |  | 0.355 | 0.322 | (0.306, 0.440) |  | 0.280 | 0.267 | (0.183, 0.411) |
| 0.45 |  | 0.312 | 0.320 | (0.079, 0.444) |  | 0.223 | 0.220 | (0.116, 0.369) |
| 0.50 |  | 0.213 | 0.205 | (0.079, 0.351) |  | 0.176 | 0.166 | (0.105, 0.315) |
| 0.55 |  | 0.179 | 0.205 | (0.085, 0.319) |  | 0.146 | 0.127 | (0.104, 0.278) |
| 0.60 |  | 0.148 | 0.140 | (0.085, 0.344) |  | 0.128 | 0.123 | (0.083, 0.198) |

**Table S2-2**. Estimated Spearman’s rho correlations between unweighted (*Q*) and weighted (*Q’*) modularity and time (in years) over various L_1_ penalty values (*ρ*). Network structures were estimated from MIC data for 16 drugs from 14,418 *E. coli* isolates collected by the FDA and USDA during 2004-12. Drug resistances were classified by the class of drug associated with resistances, and included aminoglycosides, amphenicols, β-lactams, fluoroquinolones, sulfonamides, tetracyclines, and macrolides. A decreasing trend was noted in both *Q* and *Q’* over time in all cases, but was not statistically significant in all cases.

| *ρ* |  | *Q* |  | *Q’* |
| --- | --- | --- | --- | --- |
| 0.05 |  | -0.59† |  | -0.97** |
| 0.10 |  | -0.86** |  | -0.96** |
| 0.15 |  | -0.70* |  | -0.96** |
| 0.20 |  | -0.65† |  | -0.96** |
| 0.25 |  | -0.55‡ |  | -0.93** |
| 0.30 |  | -0.39 |  | -0.89** |
| 0.35 |  | -0.71* |  | -0.83** |
| 0.40 |  | -0.62† |  | -0.80** |
| 0.45 |  | -0.69* |  | -0.80** |
| 0.50 |  | -0.81** |  | -0.83** |
| 0.55 |  | -0.89** |  | -0.52‡ |
| 0.60 |  | -0.46 |  | -0.21 |

Significance of correlation: **p < 0.01, *p < 0.05, †p < 0.10, ‡p < 0.20
