# Supplementary material for: Markov Networks of Collateral Resistance: National Antimicrobial Resistance Monitoring System Surveillance Results from Escherichia coli Isolates, 2004-2012
Source: PLoS Comput Biol. 2016 Nov 16;12(11):e1005160. doi: 10.1371/journal.pcbi.1005160 (PMC5112851; doi:10.1371/journal.pcbi.1005160)
Supplement: S3 Text — A review of the structure of the E. coli R-nets conditioned on L1 = 0.10. This penalty was identified using the objective methods described in S2, but was rejected in favor of a higher penalty due to difficulties in interpreting the dense networks produced here. Includes tables S3-1 and S3-2. (DOCX) [file pcbi.1005160.s003.docx]

**Supplement #3. Alternate R-net results.**

An alternate L_1_ penalty, *ρ* = 0.10, was identified in the process of identifying an appropriate correlation structure in the NARMS data. In general, we found these R-nets to be less useful than those presented to demonstrate the method overall. However, since networks of greater or lesser density may be preferred for different applications of the R-nets, we present here the set of networks generated by *ρ* = 0.10 as an alternate analytic scenario.

*Network structure and parameters*

The structures of *R’* are provided graphically (Fig S3). Of the 119 unique edges that could be observed, 61 edges were observed in at least one year of the study. Seventeen edges joined resistances to drugs of the same class and 44 edges joined drugs of different classes. Most of the within-class edges (14/17) were present in all 9 years of the study (Fig S4, blue areas). Among the cross-class edges, 28/44 were present in 4 years or less, and 14/44 were present in 8 or 9 years (Fig S4, red areas). Eight unique edges, observed 17 times in total, had *ω_ij_* < 0. Edges representing negative correlations were all relatively weak (|*ω_ij_*|< 0.05), were all between-class edges, and were never observed to be positive in other years. Unstable cycles were noted in *R’*_2008_ (FOX-AXO-CHL and others), *R’*_2009_ (AMI-GEN-FIS and others), *R’*_2010_ (NAL-CIP-GEN-FIS and others), and *R’*_2012_ (AZI-AMP-TET-FIS and others). The presence and strength of within- and between-class edges over time are summarized in Figures S5A and S5B, respectively.

Density varied from 31.4% (*m*_2004_ = *m*_2010_ = 33) to 39.0% (*m*_2012_ = 41). Again, no significant trend in *m̄* was noted over time (Spearman’s rho = 0.25, p = 0.51). The graph consisted of a single component in all years. The frequent dense subgraph *R*_BLA_ could be expanded to include CHL and NAL and exceed 70% density every year. Similarly, when extended to include NAL and AMI (when present) the density of *R*_AST_ exceeded 50% in all years but 2010. The vertex degrees are summarized below (Table S3-1).

Unweighted modularity ranged from *Q*_2004_ = 0.253 to *Q*_2012_ = 0.112, and weighted modularity ranged from *Q’*_2004_ = 0.441 to *Q’*_2012_ = 0.296 (Fig S6). A significant negative trend with time was noted in both *Q* (Spearman’s rho = -0.87, p < 0.005) and *Q’* (Spearman’s rho = -0.98, p < 0.005)

*Comparison to ρ = 0.25*

The structure and trends in parameters noted in the R-nets generated under *ρ* = 0.10 are similar to those generated under *ρ* = 0.25. The networks generated under the alternate penalty were 30% - 100% denser than the presented networks. The alternate R-nets contained a small number of negative partial correlations, where the presented R-nets contained only positive partial correlations. The R-nets always formed a single component given *ρ* = 0.10, while R-nets given *ρ* = 0.25 always had at least two components in addition to some drug resistances being conditionally independent of all other drugs in some years. A similar bimodal distribution of edge frequencies was noted, but was more pronounced in the R-nets generated under *ρ* = 0.10, where many more cross-class edges were observed in three or fewer years. Most observed within-class edges were still present in most years under *ρ* = 0.10, and only one additional unique within-class edge (AMI-KAN, present in 3 years) was observed. The density of the subgraphs identified by PCA were similar overall between the two penalties. The subgraph identified by *λ*_2_ which corresponded to the *R_AST_* subgraph was consistently denser under the *ρ* = 0.10 (Table S3-2). The alternate set of R-nets also included the CHL-AMI edge identified by *λ*_3, 2008_, which was absent in the presented *R*_2008_. As discussed in section 2 of this supplement, the alternate R-nets had a lower *Q* than original R-nets but *Q’* was comparable in both point estimates and decreasing trends over time.

The purpose of the L_1_ penalty is to improve the signal-to-noise ratio from a correlation or covariance matrix to improve model interpretability. The alternate L_1_ penalty, *ρ* = 0.10, was identified using AIC and appeared as an elbow in the plot of *m̄* over *ρ*. However this penalty did not appear to sufficiently reduce the ‘noise’ from large numbers of weak, transient edges. The set of R-nets presented in the primary body of the study with *ρ* = 0.25 appears to present a more parsimonious and biologically coherent model of the correlation structure of the drug resistances in this population of *E. coli*, and may be more useful for identifying potentially influential drug resistances.

**Table S3-1**. Distribution of vertex degrees in *R_y_* generated under *ρ* = 0.10. Network structures were estimated from MIC data for 16 drugs from 14,418 *E. coli* isolates collected by the FDA and USDA during 2004-12. Vertex degree describes the number of edges incident to each vertex and the number of other vertices adjacent to the vertex. Both positive and negative edges are included in the degrees below, and the presence of negative incident edges are indicated via asterisks.

| Resistance |  | 2004 | 2005 | 2006 | 2007 | 2008 | 2009 | 2010 | 2011 | 2012 |  |
| --- | --- | --- | --- | --- | --- | --- | --- | --- | --- | --- | --- |
| AMC |  | 4 | 5 | 5* | 5 | 4 | 5 | 5 | 6 | 8 |  |
| AMI |  | 3* | 4* | 4 | 4* | 4 | 6** | 1 | - | - |  |
| AMP |  | 5 | 7 | 7 | 6 | 6 | 6 | 7 | 6 | 8 |  |
| AXO |  | 5* | 4 | 5* | 4 | 5* | 4 | 4 | 4 | 4 |  |
| CHL |  | 3 | 3 | 5 | 5 | 5* | 3 | 3 | 4 | 5 |  |
| CIP |  | 1 | 1 | 1 | 2 | 1 | 2 | 3 | 2 | 2 |  |
| COT |  | 3 | 4 | 6 | 3 | 4 | 6* | 4 | 3 | 4 |  |
| FIS |  | 7** | 7** | 5 | 7** | 5 | 7** | 5* | 5 | 7** |  |
| FOX |  | 5 | 5 | 7 | 7 | 6 | 6 | 6 | 6 | 6 |  |
| GEN |  | 5* | 4 | 6** | 4 | 4 | 4 | 3 | 2 | 2 |  |
| KAN |  | 6 | 6 | 6 | 5 | 5 | 5 | 5 | 4 | 6 |  |
| NAL |  | 5* | 7* | 5 | 7* | 6 | 8** | 6* | 6 | 7* |  |
| STR |  | 5 | 5 | 6 | 6 | 5 | 6* | 4 | 5 | 5 |  |
| TET |  | 3 | 4 | 5 | 5 | 5 | 5 | 4 | 5 | 6 |  |
| TIO |  | 6 | 6 | 7 | 6 | 7 | 7 | 6 | 7 | 7 |  |
| AZI |  | - | - | - | - | - | - | - | 3 | 5* |  |

*: 1 negative incident edge (*ω_ij_* < 0); **: 2 negative incident edges

**Table S3-2**. Comparison of induced subgraph density (*m̄*, as percent) in *R*_2004_*, R*_2008_*, R*_2012_ estimated under *ρ* = 0.10 or 0.25. The subgraphs were identified by resistances that had loadings in excess of 0.4 in the PCA eigenvalues (*λ*_i_) from same Spearman’s correlation matrix **Σ** used to generate the respective *R*. The lower penalty value generated more dense subgraphs in *λ*_2_ of all years, and identified the single edge (AMI-CHL) in the *λ*_4; 2008_ subgraph, but were otherwise identical to the R-nets generated by the higher penalty.

|  |  | 2004 | |  | 2008 | |  | 2012 | |
| --- | --- | --- | --- | --- | --- | --- | --- | --- | --- |
| *ρ* |  | 0.1 | 0.25 |  | 0.1 | 0.25 |  | 0.1 | 0.25 |
| *λ*_1_ |  | 100 | 100 |  | 100 | 100 |  | 100 | 100 |
| *λ*_2_ |  | 80.0 | 73.3 |  | 86.7 | 66.7 |  | 80.0 | 53.3 |
| *λ*_3_ |  | 66.7 | 66.7 |  | 100 | 100 |  | 100 | 100 |
| *λ*_4_ |  | N/D | N/D |  | 100 | 0 |  | 100 | 100 |
