# Supplementary figures and images for: Markov Networks of Collateral Resistance: National Antimicrobial Resistance Monitoring System Surveillance Results from Escherichia coli Isolates, 2004-2012
Source: PLoS Comput Biol. 2016 Nov 16;12(11):e1005160. doi: 10.1371/journal.pcbi.1005160 (PMC5112851; doi:10.1371/journal.pcbi.1005160)

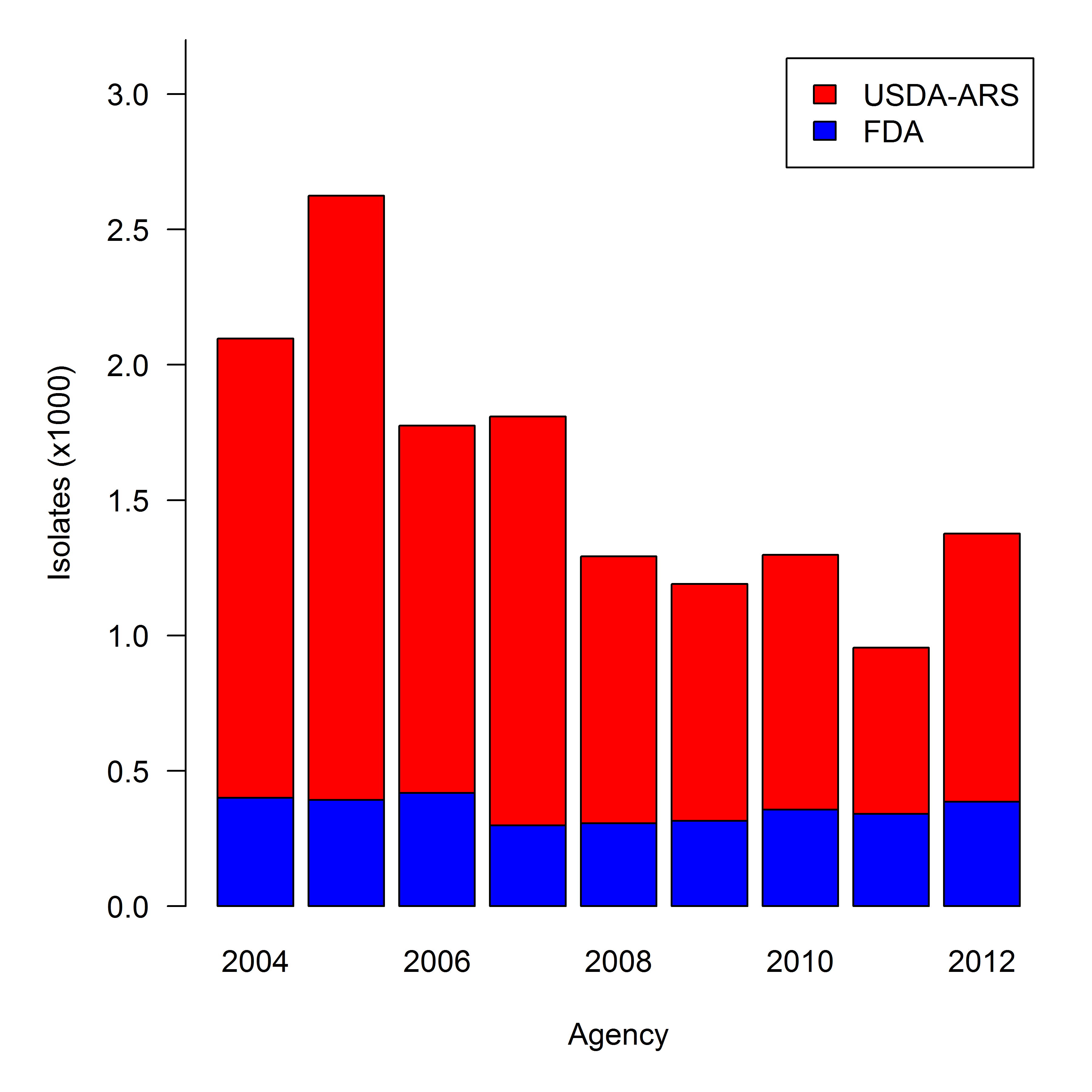

Supplement: S1 Fig — A stacked histogram representing the 14,418 E. coli isolates provided to the NARMS study between 2004 and 2012 by the United States Department of Agriculture Agricultural Research Service (USDA-ARS, red portions) and Food and Drug Administration (FDA, blue portions) from chicken carcass rinsates and commercially-packed chicken breast products, respectively. (TIF) [file pcbi.1005160.s004.tif]

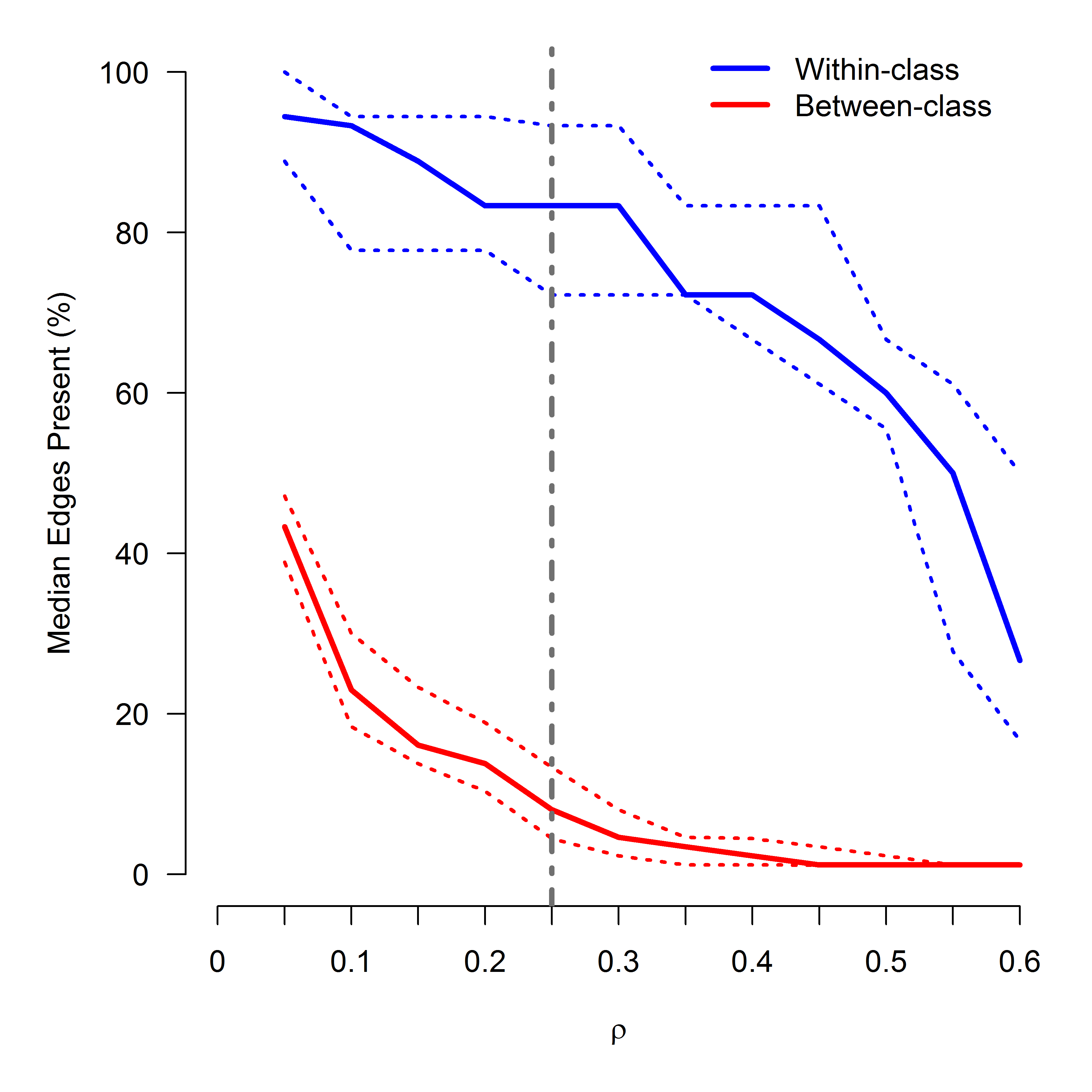

Supplement: S2 Fig — Edges joining resistances to drugs of a similar class (‘within-class’ edges) were substantially more robust to low regularization penalties (ρ < 0.30), while edges joining resistances of different antimicrobial classes were disproportionately removed by the lower penalties. The difference in behavior between these two types of edges led to differences in unweighted modularity based on the selected penalty. The drug resistances were grouped into one of the following classes based on drug structure: aminoglycosides, β-lactams, fluoroquinolones (including NAL), sulfonamides, tetracyclines, and macrolides (see Table 1). Network structures were estimated from MIC data for 16 drugs from 14,418 E. coli isolates collected by the FDA and USDA during 2004–12. The vertical line at ρ = 0.25 indicates the penalty used to generate R and R’ in the presented study. (TIF) [file pcbi.1005160.s005.tif]

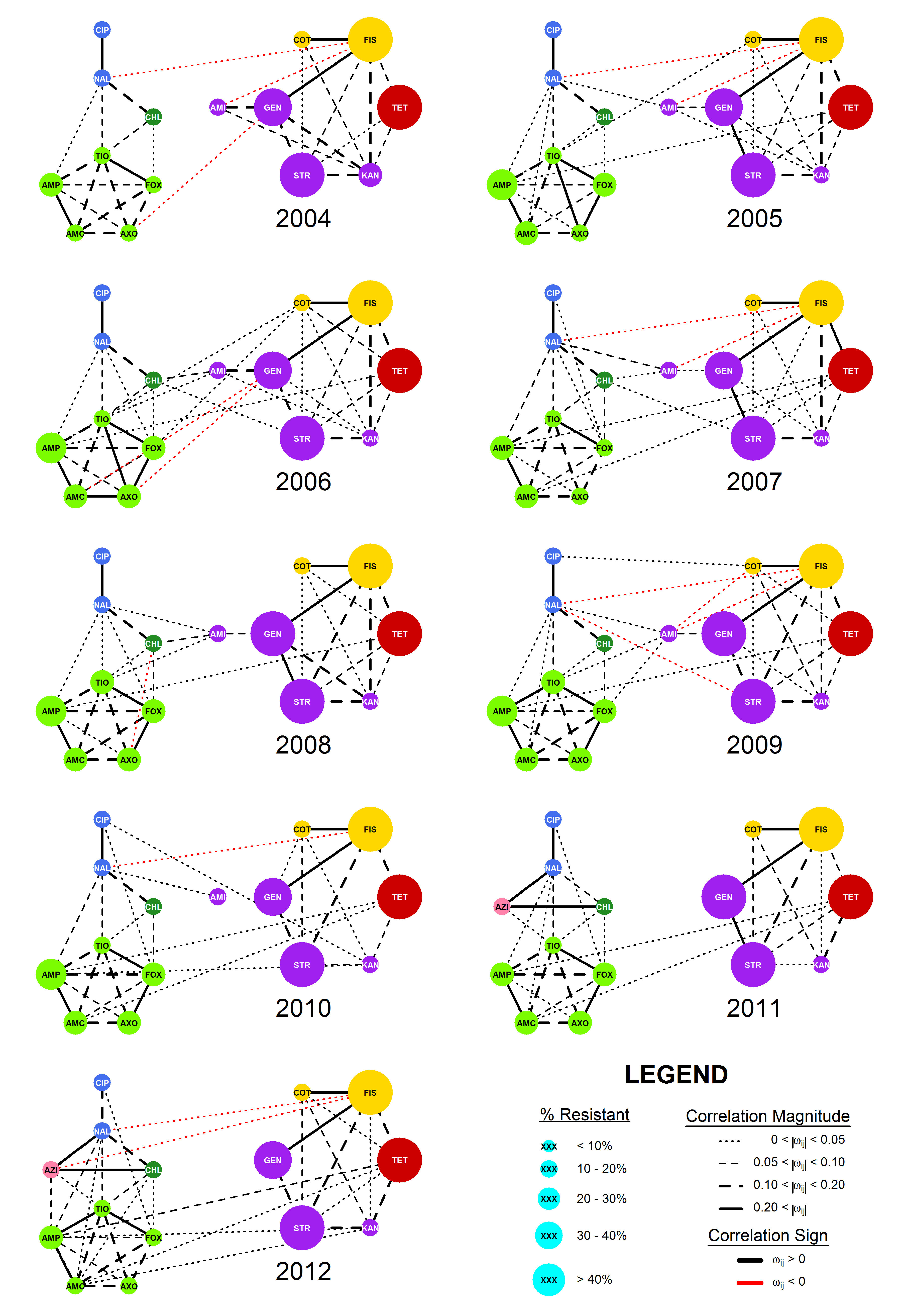

Supplement: S3 Fig — Nine weighted Markov networks representing AMR data consisting of 14,418 total isolates of E. coli collected from chicken carcass rinsates and commercial chicken breasts from each year from 2004 to 2012. Networks were generated via the graphical least absolute shrinkage and selection operator with ρ = 0.10. In general, these graphs were considered too dense to be informative and included unstable cycles in several instances. The edges were decorated with line weights and styles to indicate the relative |ωij| magnitude and the sign of the partial correlation. Vertex size indicates the percent of isolates with an MIC meeting or exceeding the published breakpoint for respective drug (See Table 2). Vertex colors indicate the Class of drug associated resistance as follows: β-lactams–light green, fluoroquinolones–blue, aminoglycosides–purple, sulfonamides–yellow; chloramphenicol–dark green; tetracycline–red; macrolide–pink. (TIF) [file pcbi.1005160.s006.tif]

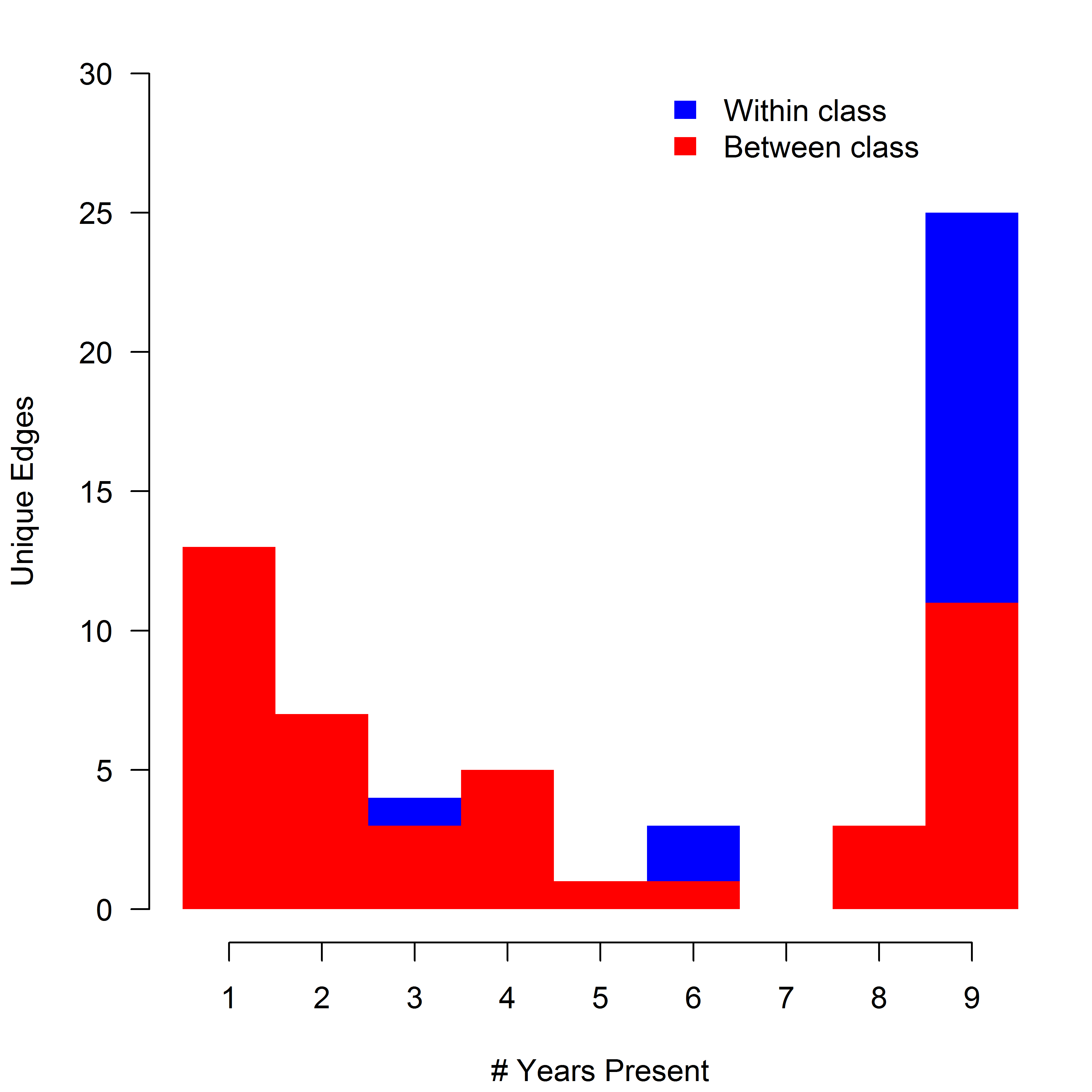

Supplement: S4 Fig — Sixty-one unique edges were identified across 9 Markov networks estimated using AMR data from 14,418 total isolates of E. coli collected from chicken carcass rinsates (USDA-ARS) and commercial chicken breasts (FDA) between 2004 and 2012. Networks were generated via the graphical least absolute shrinkage and selection operator (LASSO) with ρ = 0.10. A similar bimodal distribution to that seen under ρ = 0.25 was noted, but with a higher peak at the lower end of the plot representing infrequently present edges. Edges are categorized as either within-class (blue) or between-class (red) edges. (TIF) [file pcbi.1005160.s007.tif]

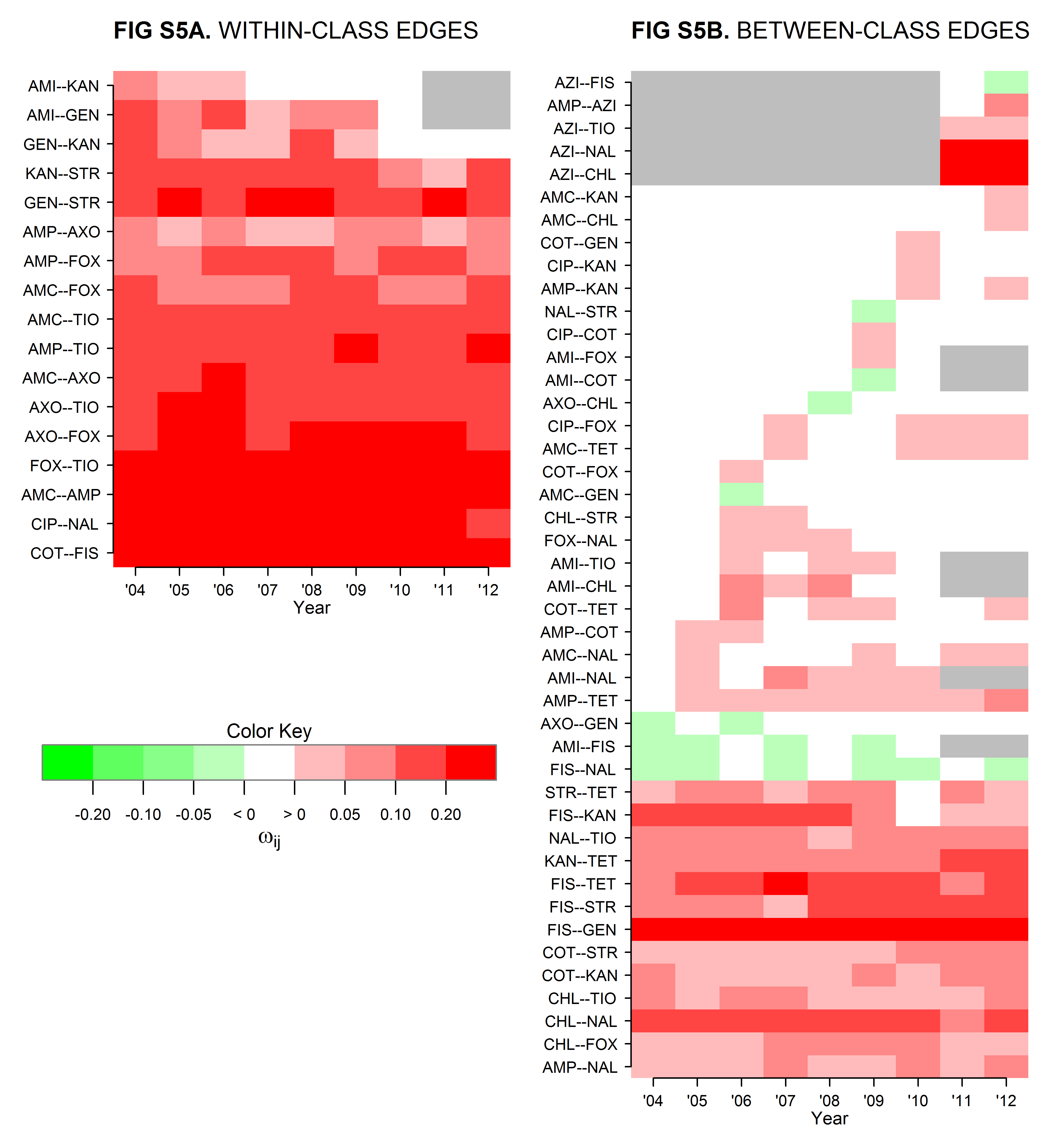

Supplement: S5 Fig — Heatmap of non-zero partial correlations between drug resistances over time under an alternate regularization penalty (A) Edge weights for 17 edges between resistances of drugs of the same class and (B) 44 edges between resistances to drugs of different classes in Markov networks of AMR data. Red and green coloring represent the magnitude of the positive and negative partial correlations, respectively, defining the edge. Grey areas represent years when one of the antibiotic drugs was not included in the panel (AMI after 2010 and AZI prior to 2011) and the edge could not be observed. The AMI-KAN edge was the only within-class edge found under ρ = 0.10 that did not appear under ρ = 0.25. The lower penalty found many more unique between-class edges (44 vs 17), but many of these were transient. Edges that appeared transiently when ρ = 0.25 were more consistently present under ρ = 0.10. (TIF) [file pcbi.1005160.s008.tif]

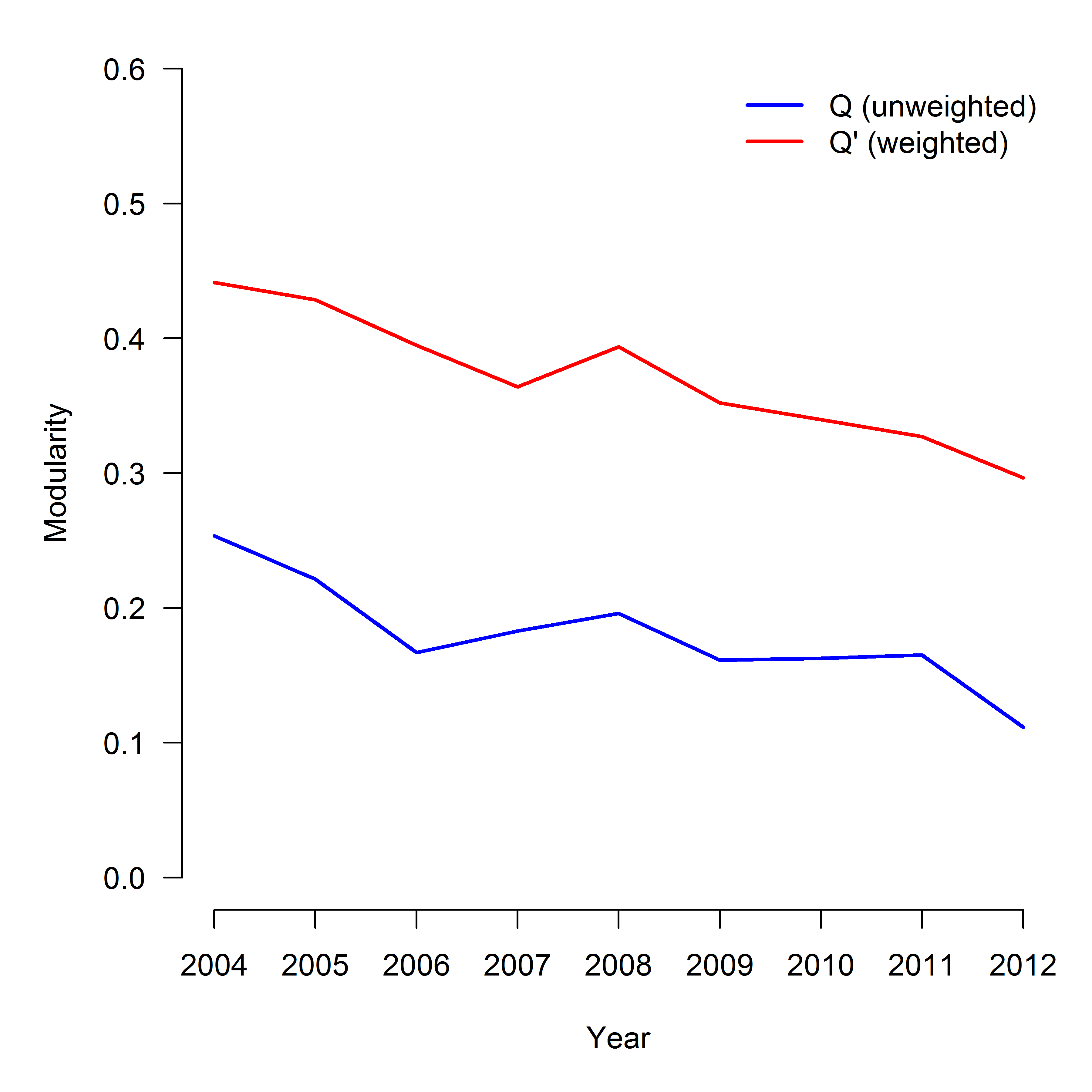

Supplement: S6 Fig — The unweighted modularity (Q, blue line) and weighted modularity (Q', red line) based on the class of drug associated with the resistance (See Table 1) for Markov networks of AMR data over time. Networks were generated via the graphical least absolute shrinkage and selection operator with ρ = 0.10. A statistically negative trend over time was noted in both Q (Spearman’s rho = -0.86, p < 0.005) and Q’ (Spearman’s rho = -0.96, p < 0.005). (TIFF) [file pcbi.1005160.s009.tiff]
